# Perioperative Levels of IL8 and IL18, but not IL6, are Associated with Nucleus Basalis Magnocellularis Atrophy Three Months after Surgery

**DOI:** 10.1007/s11481-024-10110-4

**Published:** 2024-03-14

**Authors:** Maria Heinrich, Claudia Spies, Friedrich Borchers, Insa Feinkohl, Tobias Pischon, Arjen J. C. Slooter, Clarissa von Haefen, Norman Zacharias, Georg Winterer, Florian Lammers-Lietz

**Affiliations:** 1 Charité–Universitätsmedizin Berlin, Department of Anesthesiology and Intensive Care Medicine, Corporate member of Freie Universität Berlin and Humboldt-Universität Zu Berlin, Augustenburger Platz 1, 13353 Berlin, Germany; 2 Berlin Institute of Health at Charité –Universitätsmedizin Berlin, Berlin, Germany; 3 Faculty of Health/Department of Medicine at Witten/Herdecke University, Witten/Herdecke, Germany; 4 Molecular Epidemiology Research Group, Max-Delbrück-Center for Molecular Medicine in the Helmholtz Association (MDC), Berlin, Germany; 5 Biobank Technology Platform, Max-Delbrück-Center for Molecular Medicine in the Helmholtz Association (MDC), Berlin, Germany; 6 Charité–Universitätsmedizin Berlin, Corporate member of Freie Universität Berlin and Humboldt-Universität Zu Berlin, Berlin, Germany; 7 Core Facility Biobank, Berlin Institute of Health at Charité – Universitätsmedizin Berlin, Berlin, Germany; 8 Department of Intensive Care Medicine and Brain Center, University Medical Center Utrecht, Utrecht University, Utrecht, the Netherlands; 9 Department of Psychiatry, University Medical Center Utrecht, Utrecht University, Utrecht, the Netherlands; 10 UMC Utrecht Brain Center, Utrecht University, Utrecht, the Netherlands; 11 Department of Neurology, UZ Brussel and Vrije Universiteit Brussel, Brussels, Belgium; 12 Pharmaimage Biomarker Solutions GmbH, Berlin, Germany; 13 PI Health Solutions GmbH, Berlin, Germany

**Keywords:** Nucleus basalis magnocellularis (of Meynert), Acetylcholine, Interleukins, Surgery, Atrophy, Neurodegeneration

## Abstract

Past studies have observed that brain atrophy may accelerate after surgical procedures. Furthermore, an association of systemic inflammation with neurodegeneration has been described. We hypothesize that postoperative interleukin (IL) levels in circulation as well as the perioperative change in interleukin levels are associated with increased postoperative atrophy in the Nucleus basalis magnocellularis (of Meynert, NBM) which is the major source of cortical acetylcholine. We analyzed data from the BioCog cohort which included patients ≥ 65 years presenting for elective major surgery (≥ 60min). Blood samples were taken before surgery and on the first postoperative day. Magnetic resonance imaging of the brain and neuropsychological assessments were conducted before surgery and after three months follow-up. We used linear regression analysis to determine the association of three interleukins (IL6, IL8 and IL18) with NBM atrophy (in % volume change from baseline before surgery to follow-up), as well as to examine the associations of NBM atrophy and volume with postoperative cognitive ability and perioperative cognitive change. Receiver-operating curves were used to determine the prognostic value of preoperative interleukin levels. For IL8 (N = 97) and IL18 (N = 217), but not IL6 (N = 240), we observed significant associations of higher postoperative IL levels at the first postoperative day with higher NBM atrophy at three months after surgery. Subsequent analyses suggested that in both IL8 and IL18, this association was driven by a more general association of chronically elevated IL levels and NBM atrophy, reflected by preoperative IL concentrations, rather than IL response to surgery, measured as the difference between pre- and postoperative IL concentrations. At follow-up, NBM volume was positively associated with the level of cognitive performance, but NBM atrophy was not significantly related to perioperative cognitive change. Prognostic value of preoperative IL concentrations for NBM atrophy was low. Our results suggest that an association of postoperative interleukin levels with NBM atrophy is driven by preoperatively elevated interleukins due to pre-existing inflammation, rather than perioperative change in interleukin levels in response to surgery and anesthesia. The BioCog study has been registered at clinicaltrials.gov on Oct 15, 2014 (NCT02265263).

**Graphical Abstract:**

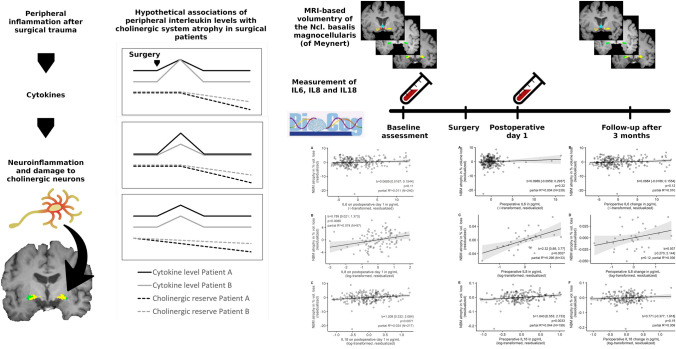

**Supplementary Information:**

The online version contains supplementary material available at 10.1007/s11481-024-10110-4.

## Introduction

Excessive perioperative inflammation is thought to be a major pathogenetic factor in postoperative neurocognitive disorders, including both delirium (POD) (Knaak et al. 2019; Lammers-Lietz et al. 2022a, b) and long-term cognitive decline (Gu et al. 2017; Kline et al. 2016; Wu et al. 2016). Whereas several studies have suggested inflammatory response to surgical trauma as a reason for subsequent neuronal damage in patients with POD, it is unknown whether or not the perioperative inflammatory reaction is a general driver of brain atrophy in surgical patients (Ballweg et al. 2021; Casey et al. 2020). Several studies describe accelerated neurodegeneration after surgery: A retrospective study reported decreased cortical thickness in patients who had undergone surgery within the prior 20 years (Sprung et al. 2019). Similarly, prospective studies described accelerated postoperative ventricular enlargement as well as increased cortical and hippocampal atrophy in surgical patients compared to non-surgical controls (Kline et al. 2012; Schenning et al. 2016). Importantly, none of these studies provides data on the association with inflammatory parameters measured during the perioperative course.

Studies in surgical patients have yielded relevant findings on interactions between acute inflammatory molecules and neuronal damage (Ballweg et al. 2021; Casey et al. 2020). The cholinergic system is thought to play a critical role in the regulation of these neurocognitive disorders. In this regard, decreased cholinergic neurotransmission is thought to promote the development of neurocognitive disorders (Hampel et al. 2018; Heinrich et al. 2020), also in the perioperative context (Bosancic et al. 2022; Heinrich et al. 2021; Muller et al. 2019).

The Nucleus basalis magnocellularis (of Meynert, NBM) is the main source of cholinergic projections to the cerebral cortex (Mesulam et al. 1983) and its role for cognition has been studied in health (Heinrich et al. 2020; Lammers et al. 2016, 2018) as well as multiple clinical conditions (Avram et al. 2021; Berlot et al. 2022; Cavallari et al. 2016; Grothe et al. 2013; Peter et al. 2016). Neuroinflammation due to intracerebral application of lipopolysaccharide has been found to lead to specific loss of cholinergic neurons in the basal forebrain of rats in a dose-dependent manner (Houdek et al. 2014). The NBM is located centrally between several circumventricular organs and may hence particularly vulnerable to blood-borne cytokines (Loy et al. 1994). To our knowledge, no study has yet investigated if the NBM is specifically vulnerable to systemic inflammation in the perioperative setting.

Postoperative IL levels integrate both preoperative IL concentrations due to chronic low-grade inflammation as well as postoperative change of IL concentrations in response to surgical trauma. We hypothesized that acute postoperative inflammation, although transient, has relevant impact on postoperative NBM atrophy due to its magnitude. We assumed that both sources (preoperative inflammation as well as response to surgery) would have additive effects best represented by absolute IL levels on the first postoperative day, and that any overshooting inflammatory response, independent of its foundation, could have deleterious effects on the brain. In our secondary hypotheses we considered that preoperative inflammation and individual immune response to surgery might have differential effects with independent contributions to brain atrophy. Hence, the aim of this study was to describe the relationship between systemic postoperative interleukin levels and NBM atrophy (as % change in NBM volume) and to determine if this association is driven by chronically elevated preoperative IL levels or perioperative change (as difference in IL levels between baseline and first postoperative day). We additionally investigated the value of interleukin levels to predict postoperative NBM atrophy and examined the associations with postoperative cognitive ability and of NBM volume with perioperative cognitive change.

## Materials & Methods

### Study Design and Procedures

This analysis is a subproject of the BioCog study (Biomarker Development for Postoperative Cognitive Impairment in the Elderly study, www.biocog.eu), which was a prospective cohort study designed to develop a biomarker-based algorithm for risk prediction of postoperative delirium and cognitive dysfunction conducted at two European tertiary care centers (Winterer et al. 2018).

Patients ≥ 65 years of European-Caucasian descent presenting for elective major surgery (≥ 60min) with MMSE score > 23 points were included. Detailed inclusion and exclusion criteria are described in a previous publication, but are also given in the online resources (supplementary A) (Heinrich et al. 2021; Lammers-Lietz et al. 2022a, b; Lammers et al. 2018, 2020).

Consenting patients underwent a preoperative baseline assessment including MRI, blood sampling, cognitive testing, and collection of various clinical data. Blood sampling was repeated on the first postoperative day. After three months, patients were invited to a follow-up assessment including MRI and cognitive testing.

For this study, we selected all patients with available longitudinal MRI data and postoperative measurements of IL6, IL8 or IL18. (For supplementary analyses in the online resource, the sample composition differed depending on the availability of additional data.) Determination of data to describe the study population has previously been published (Lammers-Lietz et al. 2022a, b; Lammers et al. 2018).

### Measurement of Interleukin Levels

Ethylenediaminetetraacetic acid (EDTA)-preserved blood samples were collected by trained clinic staff according to a standard operating procedure following overnight fasting in supine position preoperatively before induction of anesthesia as well as on the first postoperative day. EDTA tubes were placed on a rocking mixer for at least 30 s and were centrifuged at 2500G for 10 min at room temperature. Samples were frozen at -80 °C and were shipped to an analysis lab (BioVendor – Laboratorni medicina a.s., Brno, Czech Republic).

IL6 was measured with the BioVendor Human Interleukin-6 assay (Brno, Czech Republic, catalog no. RD194015200R4, detection limit: 0.65pg/mL). IL8 and IL18 were measured using a commercially available assay (ProcartaPlex Multiplex Immunoassay – Human Custom ProcartaPlex 4-plex, catalogue no. PPX-04-MXPRKHD, Thermo Fisher Scientific Inc, Carlsbad, CA, USA, measuring range: 2.25-2300pg/mL for IL8 and 14.4-14750pg/mL for IL18).

### Magnetic Resonance Imaging

Cranial magnetic resonance imaging (MRI) data were collected in two 60min scanning session with harmonized T1-weighted anatomical imaging sequences at high field strength in both study centers before surgery and during a follow-up assessment after three months. The complete neuroimaging protocol is described elsewhere (Winterer et al. 2018). In Berlin, an MPRAGE sequence (magnetization prepared rapid gradient echo in 192 sagittal slices, FOV: 256∙256mm^2^, voxel size: 1∙1mm^2^ at 1mm slice thickness, TR: 2500ms, TE: 4.77ms, 7° flip angle, parallel imaging with generalized autocalibrating partially parallel acquisitions GRAPPA using 24 reference lines, acceleration factor R = 2) was run on a 3T Magnetom Trio MR scanner (Siemens) equipped with a 32-channel head coil at the Berlin Center for Advanced Neuroimaging (BCAN). In Utrecht, an Achieva 3T MRI scanner (Phillips, Amsterdam) equipped with an 8-channel head coil was used at the beginning of the study, and later switched to an identical machine with a 32-channel head coil for technical reasons. A similar sequence was recorded (192 sagittal slices, FOV: 256∙232mm^2^, voxel size: 1∙1mm^2^ at 1mm slice thickness, TR: 2500ms, TE: 4.77ms, 7° flip angle, parallel imaging with sensitivity encoding SENSE, acceleration factor R = 2).

#### Image Processing

SPM12 (http://www.fil.ion.ucl.ac.uk/spm/software/spm12/) in a MATLAB environment (The Mathworks. Inc. Natick. MA) was used for volumetric MRI analysis with additional use of the log_roi_batch extension by Adrian Imfeld (http://www.aimfeld.ch/neurotools/neurotools.html). MR images were partitioned into grey and white matter as well as cerebrospinal fluid using the SPM12 segmentation routine. For segmentation of the basal forebrain cholinergic system (BFCS) including the NBM, a previously described probabilistic map was used. This map has been provided by Dr. Laszlo Zaborszky and was described in detail elsewhere (Zaborszky et al. 2008). At each voxel in this map, the probability of cholinergic cells was ≥ 40% based on the ten post-mortem brain specimen from which this map has been derived. Both tissue map as well as the segmented patient data were mapped onto a common template using the DARTEL (Diffeomorphic Anatomical Registration using Exponentiated Lie algebra) flow fields implemented in SPM12. These deformations were applied to the probabilistic map of the basal forebrain resulting in individual labeling of cholinergic subregions in a participant's brain scan.

The BFCS tissue map differentiates between four subregions (Ch1/2, Ch3, Ch4 and Ch4p) based on a modified version of Mesulam’s nomenclature of the cholinergic system. NBM volume was calculated as the sum of voxels in the subregions Ch4 and Ch4p. For each patient, we calculated the 3-month NBM atrophy as the ratio of volume loss and preoperative volume as previously described elsewhere: atrophy (in %) = 100∙(V_preop._–V_postop._)/V_preop_ (V: volume) (Cavedo et al. 2017).

### Cognitive Assessment

Cognitive assessments comprised the Cambridge Neuropsychological Test Automated Battery (CANTAB; CANTAB Research Suite, Cambridge Cognition Ltd., UK) which includes the Paired Associate Learning test (PAL), Verbal Memory Recognition test (VRM), Simple Reaction Time (SRT), Simple Span task (SSP) and is implemented in a touch-screen electronic device, as well as the Grooved Pegboard Test (GPT, Lafayette Instrument, Lafayette, IN, USA) and the Trail-Making Test (TMT). The cognitive testing took a total amount of 60min for completion.

Completion times of TMT Part B and the GPT, mean latency in the Simple Reaction Task (SRT), free recall in the VRM, first trial memory score from the PAL and number of remembered items in the SSP were selected as cognitive parameters of interest from the complete test battery based on their stability in a control cohort, as described elsewhere (Feinkohl et al. 2020). Detailed description of the cognitive tests is given elsewhere (Feinkohl et al. 2020; Heinrich et al. 2020; Lammers et al. 2018). Cognitive testing was performed within 14 days before surgery and at follow-up three months after surgery. We considered both postoperative cognitive performance and cognitive performance change for our analysis. Cognitive performance change was calculated as the residual of postoperative cognitive test performance regressed on preoperative cognitive test performance.

### Statistical Analysis

#### Descriptive Analyses

The sample was described using median, interquartile, and minimum–maximum ranges for continuous data, as well as absolute and relative frequencies (in %) for nominal variables. To describe associations and changes between pre- and postoperative interleukin levels, Spearman’s rank correlation coefficient (ρ) has been calculated.

#### Analysis of Postoperative Interleukin Levels and NBM Atrophy

We analyzed the association of interleukin concentration on the first postoperative day as the independent variable of interest with postoperative NBM atrophy as the dependent variable of interest in ordinary least squares (OLS) regression models with adjustment for age, sex and preoperative MMSE score and up to three dummy variables describing the four conditions for the MRI scanner hardware. The first MRI condition referred to image acquisition at the Siemens Magnetom in Berlin, the second and third conditions referred to neuroimaging at one of two Philipps Achieva in Utrecht, and the fourth condition referred to a small group of patients who switched between Philipps scanners between sessions.

Interleukin concentrations showed relevant skewness. We used logarithmic transformation to obtain closer approximations to normally distributed data. In case of data including zeros (IL6), square-root transformation was used instead.

#### Analysis of Distinct Associations of Preoperative Interleukin Levels and Perioperative Change with Cholinergic Atrophy

Perioperative change in interleukin levels and preoperative interleukin levels were added as two independent variables of interest to the OLS regression models. NBM atrophy was retained as the dependent variable of interest. Perioperative interleukin level change was calculated as the difference between logarithmic (IL8, IL18) or square-root transformed (IL6) postoperative and preoperative interleukin concentrations: perioperative change = c_postop_-c_preop_ (c: logarithmic or square-root transformed concentration). The model included NBM atrophy as the dependent variable. In addition to the perioperative change and interleukin concentration on the first postoperative day as the two independent variables of interest, age, sex, preoperative MMSE score and the previously mentioned dummy variables were included as independent variables.

#### Associations with Cognitive Outcomes

We analyzed associations of NBM and cognitive outcomes in two approaches. In a longitudinal approach, we studied the relationship of NBM atrophy and longitudinal change in cognitive performance. In a second cross-sectional approach, we studied the association of NBM volume and cognitive performance three months after surgery. In both approaches, cognitive outcomes were treated as dependent variables, and NBM volumetric variables as independent variables of interest in OLS regression models. All analyses were adjusted for age, sex, and for level of International Standard Classification of Education (ISCED levels 1–2/3–4/5–6) as done in a previous study (Lammers et al. 2018). Postoperative performance in SRT, GPT and TMT Part B underwent logarithmic transformation before analysis, whereas all other cognitive variables were approximately normally distributed.

We report regression coefficients with 95% confidence intervals for all variables of interest and partial R^2^ as a measure of effect size. The general level of significance was set to p ≤ 0.05. For our main analysis (postoperative interleukin levels), we applied Bonferroni correction (adjusted level of significance: p ≤ 0.016 [0.05/3]), but not for additional analyses, which should be therefore considered as exploratory.

#### Prediction of NBM Atrophy

We considered preoperative IL8 and IL18 levels potential prognostic markers for NBM atrophy. NBM atrophy values were transformed into a binary variable: Values > 0 were considered as atrophy. We used empirical receiver operating curves (ROC) to determine the area under the curve (AUC) with 95% confidence intervals for preoperative interleukin levels. Youden’s index was defined as the ideal cut-off value and used as a basis for the determination of sensitivity, specificity, and accuracy. For this analysis, we included all patients with available preoperative interleukin measurements irrespectively of postoperative values (see also online resources, supplementary D, table S3 for the number of available datasets).

All statistical analyses were conducted in R v4.0.3. (Bunny-Wunnies Freak Out, The R Foundation for Statistical Computing) with additional use of the quantreg, car, boot, sensemakr and ROCit packages.

Graphics were generated in ggplot2, cowplot and corrplot for R as well as Scribus v.15.2 (The Scribus Team, www.scribus.net) and GIMP v2.10 (Spencer Kimball, Peter Mattis and GIMP developer team, www.gimp.org).

## Results

### Description of the Surgical Cohort

**Fig. 1:**
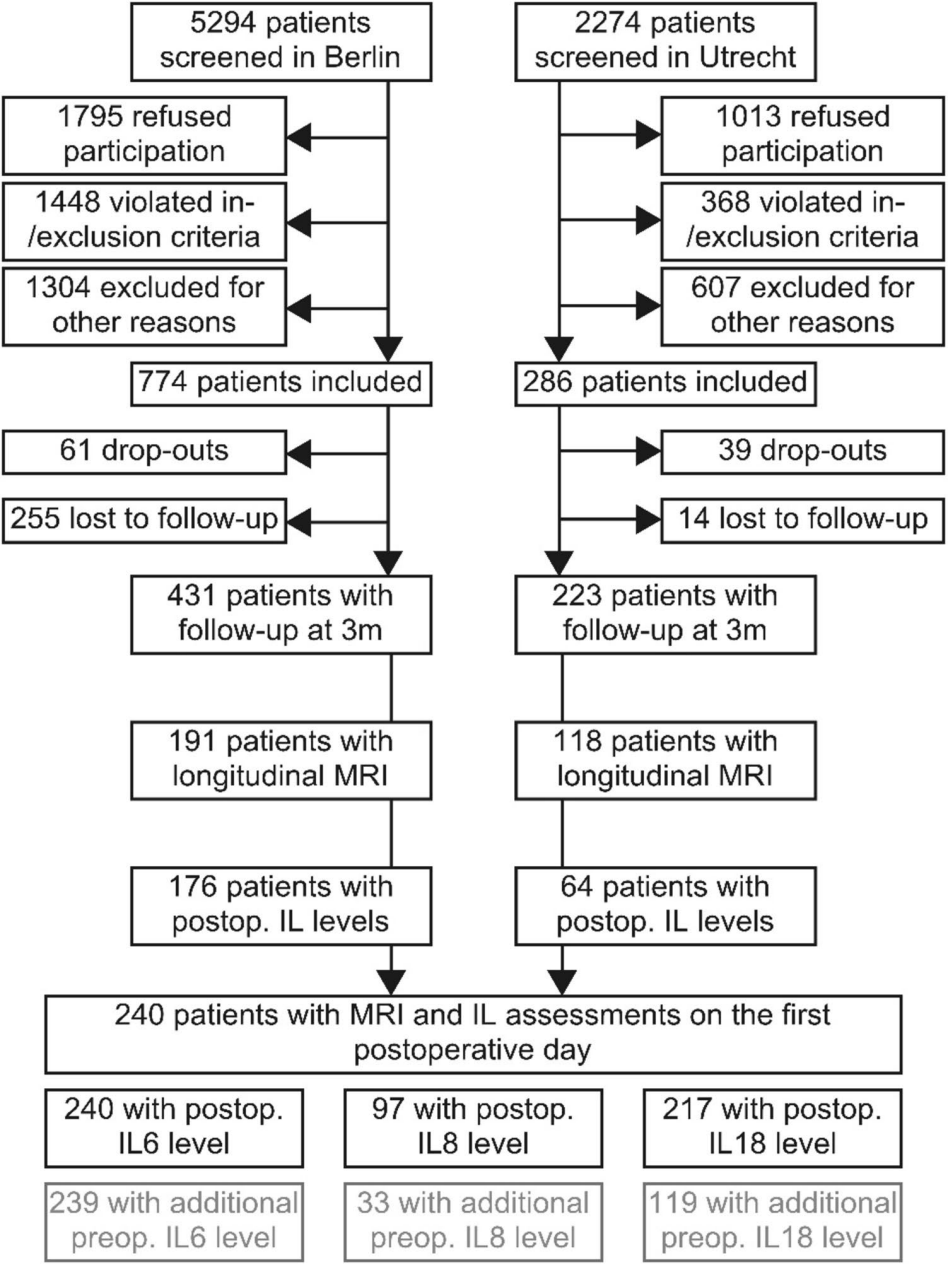
Study flow chart. Abbreviations: m – months; MRI – magnet resonance imaging; IL – interleukin

933 patients were included in the BioCog dataset. 161/247 (65%) and 321/686 patients (47%) recruited underwent MRI in Utrecht and Berlin at baseline, respectively. For 309 patients, MRI-based volumetric data of the NBM at baseline and at follow-up three months after surgery were available. For 69 patients, none of the three studied interleukins had been measured, and the final study sample comprises 240 patients with at least one available measurement on the first postoperative day (see Fig. 1 and supplementary figures S6 and S7 in the online resources). For 95 patients, levels of all interleukins were available on the first postoperative day, for N = 124 and N = 21, values of two and one studied interleukin were available, respectively. Table 1 gives a detailed description of the clinical and demographic cohort characteristics (a description of all 309 patients with longitudinal MRI is given in the online resources, supplementary table S2).

**Table 1:** Description of the study samples. Continuous variables are given as median (interquartile range) and nominal variables are displayed as absolute numbers (relative frequency in %)

		**IL6** **(N = 240)**	**IL8** **(N = 97)**	**IL18** **(N = 217)**
**Age (y)**		71 (68–75)	71 (68–75)	71 (68–75)
**Women**		82 (34%)	32 (33%)	74 (34%)
**Body mass index (kg/m**^**2**^**)**		26.4 (24.1–28.7)	27.2 (25.0–30.1)	26.5 (24.2–28.7)
**MMSE (p)**		29 (28–30)	29 (28–30)	29 (28–30)
**ASA physical score**	**I**	14 (6%)	6 (6%)	14 (6%)
	**II**	174 (73%)	59 (61%)	153 (71%)
	**III**	52 (22%)	32 (33%)	50 (23%)
**Malignant disease**		62 (26%)	32 (33%)	58 (27%)
**CCI**		1 (0–2)	1 (0–2)	1 (0–2)
**Preoperative CRP (mg/L)**		2.3 (1.0–5.9)	3.3 (1.5–7.0)	2.3 (1.0–5.9)
**Postoperative CRP (mg/L)**		28.8 (4.9–54.0)	49.4 (29.9–64.9)	30.0 (5.2–55.3)
**Preoperative leukocytes (nL**^**−1**^**)**		6.0 (5.0–7.2)	5.9 (5.0–7.1)	6.0 (5.0–7.2)
**Postoperative leukocytes (nL**^**−1**^**)**		9.3 (7.5–11.4)	9.9 (8.5–11.6)	9.4 (7.7–11.4)
**Regional anesthesia**		15 (6%)	6 (9%)	12 (6%)
**Combined regional/general anesthesia**		37 (15%)	28 (29%)	34 (16%)
**Duration of anesthesia (min)**^**a**^		176 (109–252)	221 (167–321)	176 (111–253)
**Intracavitary surgery**		101 (42%)	46 (47%)	93 (43%)
**ICU admission**		40 (17%)	20 (21%)	62 (29%)
**Length of hospital stay (d)**		4 (3–8)	7 (4–9)	5 (3–8)
**Preoperative NBM volume (cm**^**3**^**)**		1.76 (1.63–1.88)	1.77 (1.62–1.90)	1.76 (1.63–1.87)
**NBM volume loss (%)**^**b**^		0.0 (-1.8–1.8)	0.6 (1.3–2.3)	0.0 (-1.6–1.6)
**Preoperative brain volume (cm**^**3**^**)**		1009 (935–1091)	1011 (959–1102)	1010 (935–1090)
**Brain volume loss (%)**^**b**^		0.4 (-0.4–1.1)	0.6 (-0.3–1.2)	0.4 (-0.3–1.3)

N indicates the number of available postoperative datasets for each interleukin

^a^ In one study center, end of anesthesia was defined as extubation in case of postoperative prolonged ventilation, and in the other center, admission to ICU was defined as end of anesthesia, even if the patient was still intubated

^b^ Atrophy rate refers to postoperative volume loss divided by preoperative volume. Hence, positive values indicate atrophy, whereas negative values indicate that a volume gain has been measured. Please note that the minimal and maximal measured values may give a poor description of the distribution due to outliers. Reference values for a non-surgical control group are given in the supplement to guide interpretation (online resources, supplementary E). Abbreviations: y – years; MMSE—Mini-Mental State Examination; p – points; ASA—American Society of Anesthesiologists; CCI—Charlson Comorbidity Index; CRP – C-reactive protein; NBM—Nucleus basalis magnocellularis

**Fig. 2:**
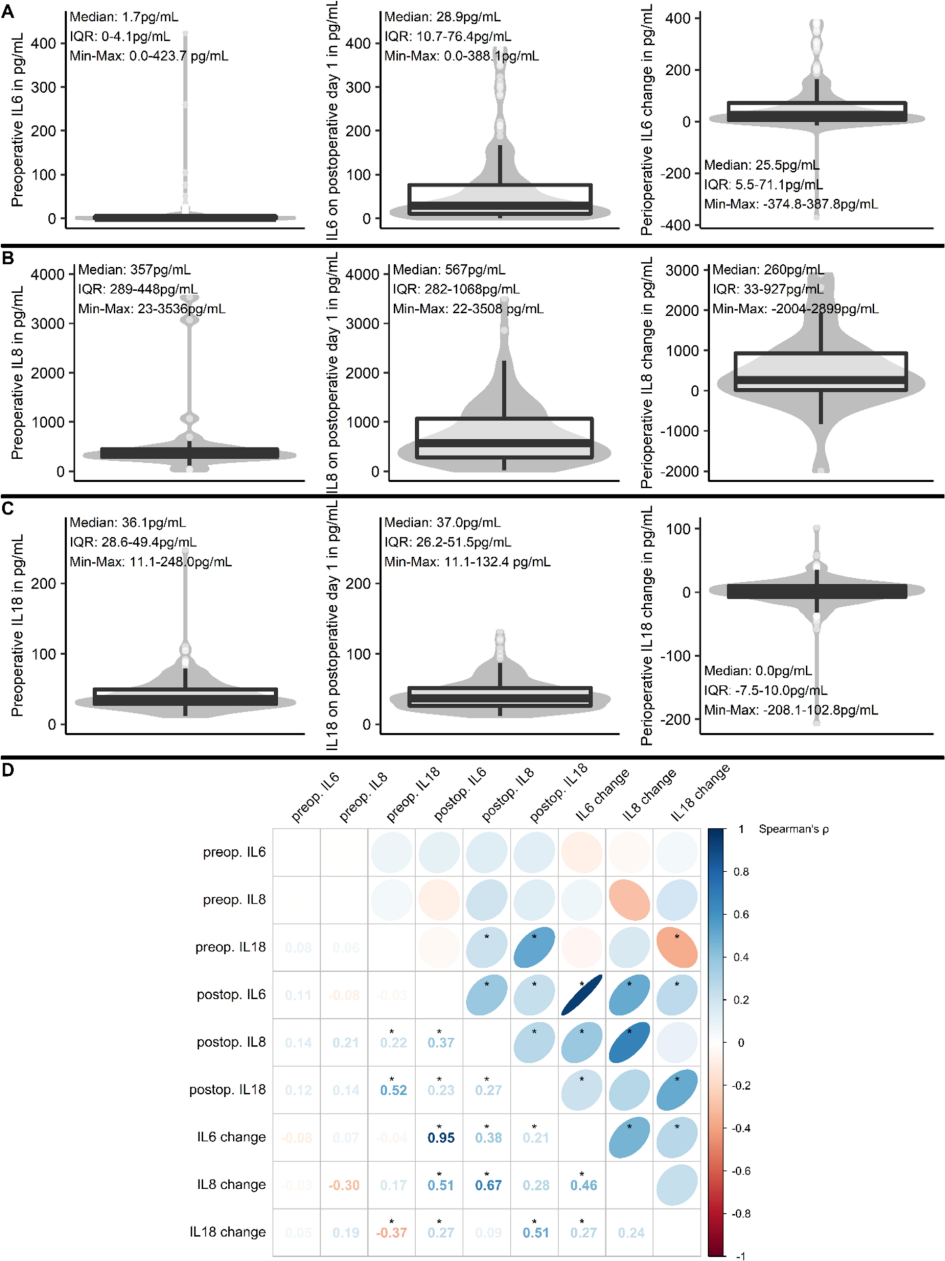
Distribution of interleukin levels and associations among interleukins. **A**, **B** and **C**: Combined box and violin plots for preoperative (left column), postoperative interleukin levels (middle column), and the perioperative change (right column). Median, interquartile (IQR) and minimum to maximum ranges are given as annotations. D: Visualized correlation matrix for interleukin levels and changes. Values, shape in the matrix and color legend correspond to Spearman’s rank correlation coefficient ρ (circle: ρ = 0, ellipse: ρ approaches 1/-1; dark blue: strong positive correlation, dark red: strong negative correlation). Significant associations with p < 0.05 have been flagged with an asterisk (*). Abbreviations: IL – interleukin; preop. – preoperative; postop. – postoperative

Figure 2A-C shows pre- and postoperative concentrations as well as change in interleukin concentrations: In all interleukins, perioperative changes showed high interindividual variability. For IL6 (Fig. 2A) and IL8 (Fig. 2B), a net postoperative increase of interleukins was observed at the median, whereas IL18 levels (Fig. 2C) did not show an overall change. In addition, pre- and postoperative IL18 values showed a strong correlation. In all interleukins, a negative correlation of preoperative values and perioperative change was observed: In patients with higher preoperative interleukin levels, a dampened postoperative increase or even decrease was observed. This finding was most prominent for IL18 (see Fig. 2D, for details).

### Association of Postoperative Interleukin Response and Cholinergic Atrophy Three Months after Surgery

**Fig. 3:**
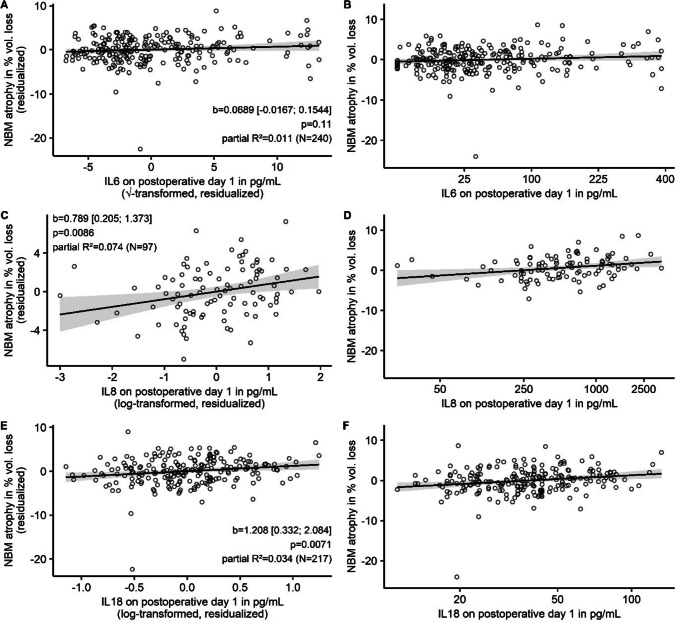
Partial regression plots (**A**, **C** and **E**) and simple scatter plots (**B**, **D** and **F**) for the association of postoperative interleukin levels (on the abscissa) with NBM atrophy (in % loss of preoperative volume on the ordinate). Higher positive values correspond to more advanced atrophy. The regression line is derived from OLS regression. 95% CIs are displayed as gray shading around the regression lines. Partial regression plots (left) means that native variables have been replaced by residuals of the variables after regression on confounders to remove their influence, explaining negative values. Regression coefficients refer to the amount of volume loss after three months in % per one unit change of the transformed IL concentration. Hence, for IL6, the b coefficient describes the estimated volume loss for an increase in IL6 concentration (c) from c1 =  × 2 to c2 = (x + 1)2. For IL8 and IL18 the % volume loss is given for an increase in concentrations by factor of approximately 2.72 (Euler’s number). For comparison of associations with NBM atrophy between three interleukins, simple scatter plots with identical scaling of the ordinate are given on the right. On the abscissa, scales have been transformed, but values refer to the actual measured IL plasma levels in pg/mL

Higher postoperative levels of IL8 and IL18, but not IL6 were significantly associated with increased NBM atrophy after surgery (see Fig. 3 for details on the statistical results and an illustration). In numbers, for an increase of IL8 or IL18 on the first postoperative day by a factor of 2.72 we observed a volume loss of 0.79% and 1.21%, respectively. Supplementary results from quantile regression yielded comparable regression coefficients (see online resources, supplementary B, table S1, models 1–3, and supplementary Fig. S1). The effects were specific for the NBM and not observed for global brain atrophy. Furthermore, the association was specific for interleukin levels and not related to clinical surrogate parameters for the extent of surgery, such as duration of anesthesia and need for ICU admission (see online resources, supplementary C).

### Distinct Associations of Preoperative Interleukin Levels and Perioperative Change with Cholinergic Atrophy

**Fig. 4:**
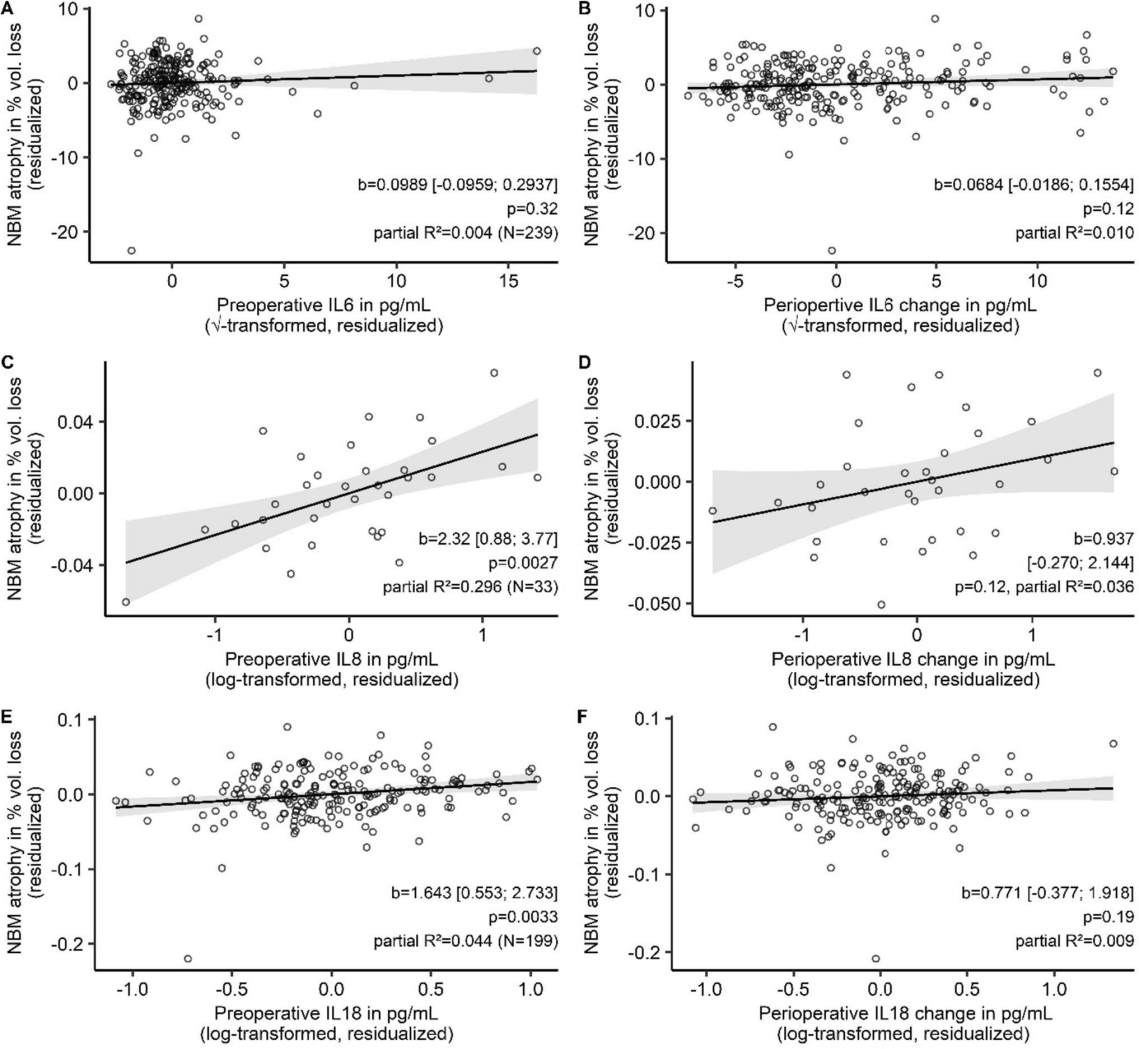
Partial regression plots for preoperative interleukin levels (**A**, **C** and **E**) and perioperative increase (difference between levels on the first postoperative day and preoperative level: **B**, **D** and **F**). Please refer to Fig. 3 for additional information. Corresponding simple scatter plots with native (not residualized) variables is given in the online resources (Figure S2). Abbreviations: NBM—Nucleus basalis magnocellularis (of Meynert); IL – interleukin

Significant associations of preoperative cytokine levels with NBM atrophy after three months were found for both IL8 (Fig. 4C) and IL18 (Fig. 4E). In numbers, an increase in preoperative interleukin levels by a factor of 2.72 was associated with a volume loss of 2.3% for IL8 and 1.6% for IL18, respectively. For neither of these two cytokines, perioperative change in concentrations was associated with atrophy (Fig. 4D + F). Neither preoperative levels nor perioperative change in IL6 levels (Fig. 4A + B) were significantly associated with NBM atrophy (see Fig. 4 for details of the statistical results). In general, quantile regression yielded similar regression coefficients (see online resources, supplementary B, Table S1, model 4–6 and Fig. S2). Multicollinearity was not critical (see online resources, supplementary D, Table S3).

### Associations of NBM Volume and Atrophy with Cognitive Outcomes Three Months after Surgery

NBM atrophy was not significantly associated with cognitive performance change. Higher postoperative NBM volume was significantly associated with better postoperative cognitive performance in the VRM free recall task (B = 1.88 [0.70; 3.06], p = 0.0019, partial R^2^ = 0.034) and completion time for Part B of the TMT (B = -0.26 [-0.52; -0.01], p = 0.0417, partial R^2^ = 0.016, see online resources, supplementary F, tables S5 and S6).

### Predictive Value of Preoperative Interleukins for NBM Atrophy

Based on Youden’s index, 524pg/mL was the ideal cut-off value for IL8 (accuracy: 61.7%, sensitivity: 32.0%, specificity: 95.5%) and 49.4pg/mL for IL18 (accuracy: 60.7%, sensitivity: 37.8%, specificity: 84.3%). The AUC for preoperative IL8 was 0.64 (0.49; 0.80) and 0.59 (0.52; 0.67) for IL18.

## Notes

Here, we analyzed the association of postoperative interleukin levels as well as the perioperative change in interleukin levels with atrophy of the NBM three months after surgery. In summary, we observed that higher levels of IL8 and IL18 on the first postoperative day, but not IL6, were associated with increased atrophy of the NBM. This finding was specific for the cholinergic system, and similar associations were not observed for whole brain atrophy rates (see online resource, supplementary C). As previously done in other studies (Cavedo et al. 2017; Kant et al. 2023), percentage changes were preferred over absolute volume changes for definition of NBM atrophy since we could not exclude baseline NBM volume to affect atrophy: On the one hand, pre-existing cholinergic atrophy may increase susceptibility for inflammatory damage (Field et al. 2012), but on the other hand, the NBM is a small region including various non-cholinergic structures, suggesting that postoperative loss of cholinergic neurons may have less impact on absolute decreases in volume as cells could be clustered more densely in patients with small NBM volume or in patients with very few cholinergic cells at baseline due to preoperative neurodegeneration (Mesulam et al. 1983; Zaborszky et al. 2008).

IL8 is a neutrophile attracting chemokine, which is a known to pass the blood–brain barrier in inflammation (Narita et al. 2005). Earlier works already hypothesized that cerebral IL8 might misguide neutrophiles, leading to immune cell invasion into neuronal tissue, explaining a variety of findings on associations between IL8 and cognitive impairment (Ballweg et al. 2021; Baune et al. 2008; Casey et al. 2020).

IL18 has various physiologic effects on a wide range of immune cells including microglia and may hence trigger neuroinflammatory processes (Prinz & Hanisch 1999; Yasuda et al. 2019). As pro-IL18 is activated by caspase-1, it is a surrogate marker for inflammasome activity and innate immune system response (Yasuda et al. 2019).

**Fig. 5:**
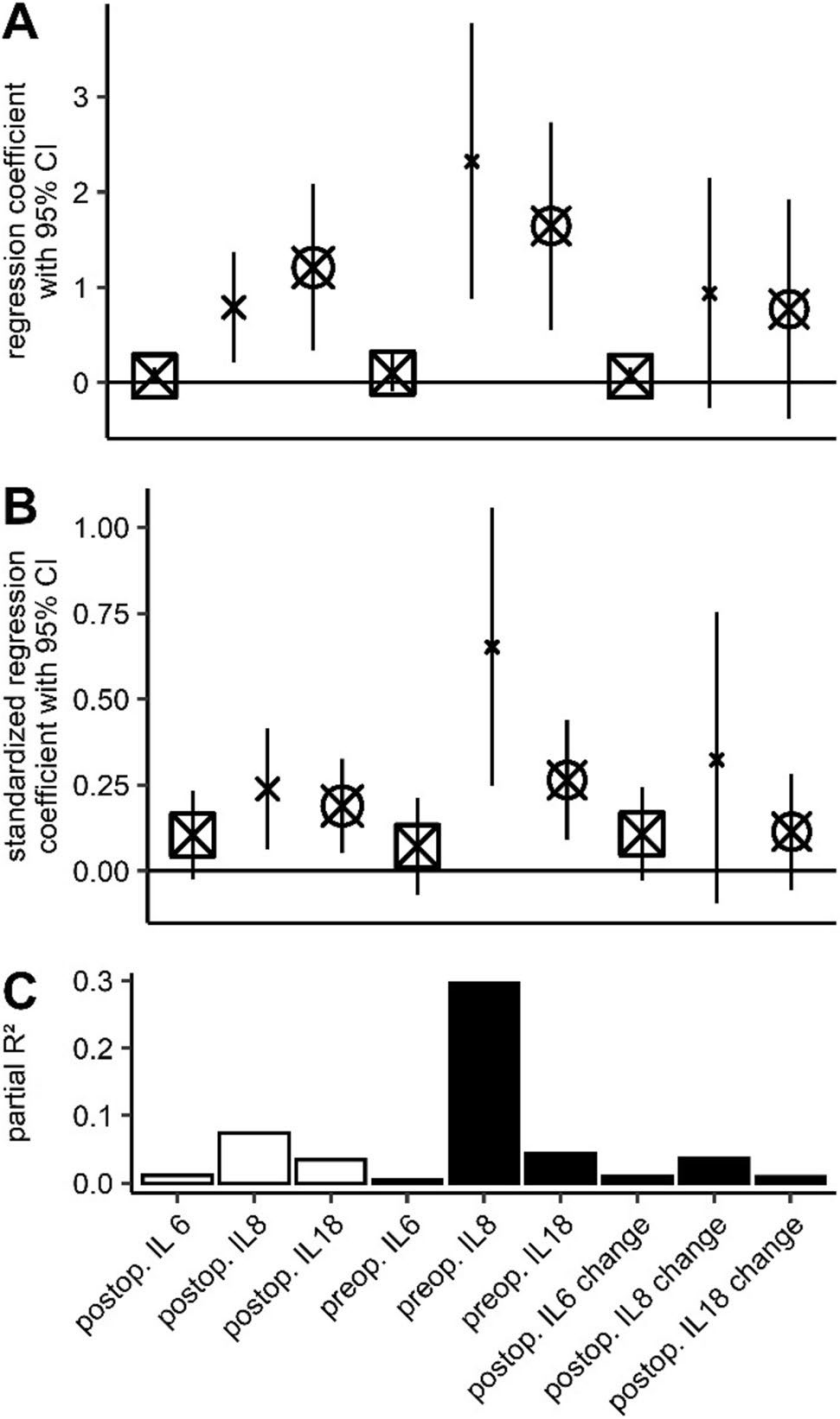
Summary of effect sizes. Plots A and B show regression coefficients (x) with 95% confidence intervals (whiskers), with cross size reflecting sample size. In A, values correspond to unstandardized regression coefficients (as reported Figs. 3 and 4), whereas in B, standardized regression coefficients are given. Standardized regression coefficients describes by how many standard deviations the value of the dependent variable will change if the value of the independent variable is increased by one standard deviation. Bar chart C displays partial R2 for all analyses, reflection the fraction of variance in NBM atrophy which is explained exclusively by interleukin levels or their perioperative change, respectively. Legend: Boxed crosses correspond to IL6, unboxed crosses to IL8, and circled crosses correspond to IL18. White bars correspond to results presented in Fig. 3, whereas black bars represent results displayed in Fig. 4

Literature suggests that beyond neuronal damage due to acute systemic inflammation, chronic low-grade inflammation leads to progressive neurodegeneration and brain atrophy (Gu et al. 2017; McCarrey et al. 2014; Su et al. 2019). Hence, we decided to adjust for preoperative interleukin levels in our analysis of acute-on-chronic effects for two reasons: First, we considered preoperative inflammation a significant confounder, since chronically elevated inflammatory cytokines may influence the perioperative inflammatory reaction to surgical trauma. Second, we assumed that low-grade preexisting inflammation and inflammatory reaction to surgery may have additive effects. However, after discarding absolute postoperative interleukin levels from the models and adding preoperative levels and perioperative change in interleukin concentrations, we observed that the association was primarily driven by pre-existing elevation of interleukins before surgery, rather than perioperative immune reaction dynamics. For comparative purposes, we have summarized the effect sizes of our analyses in Fig. 5. Of note, preoperative levels of IL8 and IL18 explained almost 30% and 4.4% of postoperative change in NBM volume, respectively, whereas postoperative change explained 3.6% and less than 1%. To our knowledge, this is the first study reporting an association of perioperative inflammation and subsequent brain atrophy. Previous studies either compared atrophy between surgical and non-surgical cohorts without measuring inflammatory parameters (Kline et al. 2012; Schenning et al. 2016; Sprung et al. 2019), or described associations of inflammation and atrophy in non-surgical patients (Gu et al. 2017; McCarrey et al. 2014).

In a previous study, Sanders et al. used neurofilament light (NfL) as a blood-sampled brain-derived biomarker of postoperative neuronal damage in a sample of surgical patients. They observed correlations between the perioperative rise in NfL as a measure of neuronal damage and three interleukins (IL1β, IL8 and IL10) (Casey et al. 2020). In another report, the same study group described an association of perioperative changes in plasma tau protein as biomarker of axonal damage and IL8 as well as IL10 (Ballweg et al. 2021). Our study extends beyond this finding by showing how pre- and postoperative interleukin levels relate to midterm brain atrophy in MRI, and further points towards a specific vulnerability of cells in the NBM (see online resources for comparison with brain atrophy). Notably, Sanders’ analyses favor perioperative immune reaction as the driving factor, whereas our analyses suggest that pre-existing inflammation is more crucial (see online resources for a comparative analysis of clinical surrogate parameters for the extent of surgical intervention).

The association of a single, isolated measurement of an inflammatory parameter as an indicator of chronic inflammation with neurodegeneration has been investigated previously. Gu et al. analyzed IL6, CRP and α1-antichymotripsin levels in 306 non-demented community-dwelling participants with a mean age of 81 years who underwent longitudinal neuroimaging with a follow-up time of 2–7 years but observed no effect of inflammatory biomarkers on hippocampal atrophy (Gu et al. 2017). McCarrey et al. studied 121 cognitively normal adults from the BLSA neuroimaging study with a mean age of 69 years. They observed significant effects of baseline IL6 levels on longitudinal cortical thinning in widespread brain areas (McCarrey et al. 2014). Our results are similar to the observations by McCarrey, since preoperative inflammation seemed to be more important than postoperative interleukin reaction. Indeed, median atrophy were comparable between our patient cohort and non-surgical controls (see Table 1 and online resources, supplementary C, Table S2 and supplementary E, table S4), suggesting that our findings also reflect a more general association between chronic inflammation and brain atrophy rather than a surgery-related process. On the other hand, inter-individual variation in atrophy was slightly higher in surgical patients, and it is unknown if preoperative increased peripheral immune activation triggered neuroinflammatory processes we have not captured in our study, explaining the more diverse outcomes in surgical patients.

In our study, there was no association between NBM atrophy and perioperative change in cognitive performance, but we observed significant cross-sectional associations between postoperative NBM volume and cognitive performance at follow-up, comparable to our previously reported findings on basal forebrain cholinergic system volume and cognition in preoperative surgical patients (Heinrich et al. 2020; Lammers et al. 2018). Although postoperative NBM volume explained only a small fraction of the total performance in the TMT-B (3.4%) and VRM (1.6%), our results point towards some clinical and functional relevance of inflammation and cholinergic atrophy for cognition in general, but our data do not support the hypothesis that postoperative inflammation accelerates cognitive decline due to loss of neurons in the NBM.

We noticed that in our data, both NBM and brain volume measurements were contaminated by outliers, and carefully assessed if they would affect the reliability of our results. Furthermore, we observed that median brain atrophy was 0.4% until follow-up and showed little interindividual variation. Hence, our findings were comparable to annual atrophy rates reported in community-dwelling participants (Gu et al. 2017; McCarrey et al. 2014). A recent publication by Kant et al. reporting brain and grey matter atrophy rates in surgical patients as well as non-surgical control participants reported similar atrophy rates (i.e., 0.3% of postoperative brain volume loss) including volume gain (i.e., 0.2% of postoperative brain volume gain in non-surgical patients) (Kant et al. 2023).On the contrary, median NBM atrophy was 0.0% with wider interindividual variation, including volume gain (see Table 1). Although the mechanisms behind this observation will remain unclear, we need to comment this finding. First, the measurement of NBM volume is more complex and requires more assumptions to be met. Hence, we cannot exclude that measurement error is considerably higher for NBM volumetry. On the other hand, multiple mechanisms may cause a postoperative gain of NBM volume: These include edema and cell swelling as a sign of neuroinflammation, compensatory hypertrophy as well as regenerative processes. Inflammatory cell swelling seems unlikely as one would expect higher interleukin levels to be associated with volume gain. Compensatory hypertrophy of cholinergic cells has previously been discussed in the context of lesion studies in animals (Pearson et al. 1984, 1985, 1987) and more recently after findings of septal nuclei enlargement in healthy adults who later developed Alzheimer’s disease (Butler et al. 2018). Postoperative regeneration of brain tissue has been observed in single studies on specific procedures, e.g., in patients with chronic sinusitis (Whitcroft et al. 2018) and cataract (Lou et al. 2013) as well as after bariatric surgery (Gabay et al. 2018; Rullmann et al. 2019). Similar changes had been described after cardiovascular rehabilitation (Anazodo et al. 2013) and training programs reflecting the motor-sensory enrichment of patients after successful surgery (Rehfeld et al. 2018).

Since we analyzed logarithmically transformed concentrations of IL8 and IL18, regression coefficients in our models need to be interpreted as atrophy rates per 2.72-fold change in interleukin levels. Hence, a 2.72-fold increase of IL8 and IL18 on the first postoperative day led to an additional postoperative NBM volume loss of 0.79% and 1.21%, and for preoperative interleukin levels, this increase was associated with an additional volume loss of 2.3% for IL8 and 1.6% for IL18, respectively. Notably, the interquartile range for preoperative interleukin levels covered ranges of 1.5- (IL8) and 1.7-fold change (IL18), and even ranges of 3.8- (IL8) and 2.0-fold (IL18) changes for postoperative interleukin levels, suggesting that interleukin ranges in the perioperative setting are associated with additional NBM atrophy in the range of 0.4–3.2% volume loss. These values are large compared to normal NBM atrophy (median 0.1% volume gain after three months, see online resources, supplementary table S4) and cortical atrophy (mean 0.2% postoperative volume gain after three months, as reported by Kant) observed in a control group of non-surgical participants from the same study (Kant et al. 2023). In comparison, Cavedo and colleagues reported annual atrophy rates of 0.74% for the cholinergic system in the basal forebrain of patients with placebo-treated prodromal Alzheimer’s disease (Cavedo et al. 2017).

Based on our results from the analysis of acute on chronic effects, we hypothesized that preoperative IL8 or IL18 levels could have prognostic value for NBM atrophy. However, discrimination was poor. We think that our results have clinical relevance since they suggest a pathomechanistic link between inflammation and neurodegeneration in surgical patients. Although we have not addressed the association of delirium with postoperative atrophy of the NBM here, Kant et al. reported increased cortical atrophy in patients with delirium from the same cohort (0.5% after three months compared to 0.2% in patients without delirium) (Kant et al. 2023). It is known that regional atrophy of the cholinergic system in the basal forebrain is associated with regional atrophy of the cortex depending on their structural connectivity (Kilimann et al. 2017; Teipel et al. 2016). However, our supplementary analyses of associations between interleukin levels and global brain atrophy suggest a specific susceptibility of the cholinergic system to inflammation (see online resources, supplementary C). Several studies, including our own work, have already highlighted the role of IL8 in postoperative delirium (Ballweg et al. 2021; Casey et al. 2020; Lammers-Lietz et al. 2022a, b). However, the relevance for cholinergic atrophy in delirium and a potential causal role of IL8 need to be determined. Currently, evidence points towards the cortex as the main site of delirium-related brain damage (Kant et al. 2023; Racine et al. 2020).

IL8 is a widely abundant chemokine guiding neutrophils to the inflammation site (Leonard & Yoshimura 1990). Baune and colleagues previously described a specific association of IL8 and poor cognitive performance and hypothesized that, since IL8 has been found to pass the blood–brain barrier, may cause peripheral inflammatory processes to shift into the brain (Baune et al. 2008; Narita et al. 2005). Several IL8-targeting agents have been described: PA401 is recombinant IL8 with increased binding affinity to glycosaminoglycanes but with no activity on the IL8 receptor. PA401 has been designed to oust IL8 from binding to endothelial cells and was found to reduce LPS-induced lung inflammation (Adage et al. 2015). Furthermore, the efficacy and safety of two anti-IL8 antibodies, ABX-CXCL8 and HuMax-IL8/BMS-986253, has been studied in COPD and metastatic or unresectable solid tumors (Bilusic et al. 2019; Mahler et al. 2004). To our knowledge, none of these agents has been studied for neurodegenerative disease or for perioperative application.

IL18 has various physiologic effects on a wide range of immune cells (Yasuda et al. 2019). It may be of particular relevance for postoperative neurodegeneration since, apart from immune and other peripheral cells, IL18 is produced by cerebral microglia (Conti et al. 1999). Furthermore, microglia respond to stimulation by IL18, and its mRNA can be detected in brains of rats which were killed by anesthesia without previous stimulation of the immune system (Prinz & Hanisch 1999; Wheeler et al. 2000). Furthermore, IL18 plasma levels as well as mRNA brain tissue levels are increased in patients with AD (Malaguarnera et al. 2006; Ojala et al. 2009). Anti-IL18 antibodies are available as a potential pharmacological approach as well as recombinant IL18 binding protein but have not yet been investigated for neurodegenerative disease (McKie et al. 2016; Gabay et al. 2018; Mistry et al. 2014).

Of note, we observed no association of IL6 and NBM atrophy, in contrast to reports in a non-surgical sample (McCarrey et al. 2014). IL6 has been found to trigger neuroinflammation and pass the blood–brain barrier (BBB), and hence would have been a candidate mediator of peripheral inflammation into the central nervous system (Banks et al. 1994; Spooren et al. 2011). In AD patients, a strong association of IL6 levels in blood and cerebrospinal fluid has been observed, suggesting that peripheral IL6 may reflect cerebral immune reaction. However, the study same did not find evidence for an association between IL6 levels and BBB permeability, suggesting that peripheral inflammation itself is not the cause of BBB leakage (Sun et al. 2003). Our study was not able to corroborate the hypothesis that peripheral IL6 drives NBM atrophy.

### Strengths and Limitations

The BioCog project offered ideal conditions to investigate the effect of inflammation on neurodegeneration in the human organism since the immune reaction is bound to a well-defined and prospectively known trigger: surgery. This circumstance is further complemented by a sufficiently long follow-up time and the availability of an MRI-derived measure of brain tissue loss, which can be considered a temporally stable biomarker for neurodegeneration. The variety of blood-sampled biomarkers collected in the BioCog study further allowed to focus this study on inflammatory pathways which can be used for pharmacological interventions in the future. However, we also have to acknowledge some limitations. In our data, NBM atrophy measurements have been contaminated with outliers. Nevertheless, quantile regression has reproduced findings from ordinary least squares regression, and hence, we consider our results as reliable.

Generally, we found that the associations we observed had effect sizes at a clinically relevant level. However, the functional relevance of cholinergic atrophy does not only depend on the atrophy rate, but also on baseline volume. I.e., a patient with a large cholinergic reserve before surgery may not suffer from postoperative cognitive impairment although a significant number of cells in the NBM had been lost.

The number of complete datasets with pre- and postoperative measurements was low, especially for IL8, limiting the reliability of effect sizes. Hence, the extremely large effect size of preoperative IL8 may be overestimated, whereas on the other hand, the model may have been underpowered to show a significant association of perioperative increase in IL8. In addition, demographic and clinical characteristics of the subsample with IL8 data suggested that these patients tended to have a higher morbidity with more extensive interventions. We ran additional analyses to assess selection bias in the association of IL8 and NBM atrophy (online resources, supplementary G) which did not suggest an overestimation of the effect size. However, we observed that the strength of association for IL18 and NBM atrophy was lower in a subsample of patients with available IL8 data, suggesting that an effect of IL18 on postoperative cholinergic neurodegeneration might be more relevant in samples with well-preserved global physical status and minor surgical interventions. Please note that a sample of patients who were willing to return for MRI three months after surgery can be expected to have a higher functional status and more favorable surgical outcomes compared to the complete study cohort which might introduce selection bias. Whereas the association of IL8 and NBM atrophy seems reliable in this respect, the association with IL18 might not be generalizable to samples with high morbidity and extensive surgical interventions.

And finally, we expected pre- and postoperative interleukin levels to be correlated and therefore included perioperative change in interleukin levels as a variable in the model. Of note, preoperative levels and perioperative change were still not completely independent since preoperatively elevated levels seemed to be associated with a dampening of the perioperative increase. Hence, it is difficult to differentiate between effects of pre- and postoperative inflammatory mediators, although calculation of variance inflation factors and additional analysis did not indicate critical collinearity of both parameters (online resources, supplement C). Of note, a plethora of interleukins has been analyzed in the BioCog study, which have not been studied here due to an insufficiently low number of available datasets for the first postoperative day among patients with longitudinal MRI assessments. Finally, our analysis provides no data on the pathway by which peripheral cytokine levels modulate neurodegenerative processes. The BioCog study did not provide data of acute neuronal death (e.g., neurofilament light protein) or microglia activation (e.g., sTREM2), which could help to elucidate molecular mechanisms, and hence, further studies are needed. In this study, postoperative IL levels were treated as an exposure and NBM atrophy as the outcome. Of note, baseline NBM volume was measured before surgery, so before measurement of the exposure factor. Although this might be considered a source of bias, we like to emphasize that the interval between preoperative MRI and surgery was generally short (within 14 days prior to surgery, usually on the day before surgery), and hence the amount of atrophy occurring between preoperative MRI and surgery is neglectable.

## Conclusion and Future Perspective

In conclusion, this is the first report on associations of perioperative interleukin levels with atrophy of the NBM. We therefore describe a pathomechanism which bears a potential molecular target for pharmacological modulation of neurodegeneration in postoperative neurocognitive disorders. We believe that our study may serve as a starting point for future studies on cholinergic atrophy and interleukin-targeting therapies in cognitive decline.

## Supplementary Information

Below is the link to the electronic supplementary material.

Supplementary file1 (PDF 6943 KB)

## Data Availability

The data presented here is not publicly available as due to the nature of this research, participants of the original study did not agree for their data to be shared publicly. Requests for data access can be send to Prof. Spies (claudia.spies@charite.de) and may be granted in accordance with legal data protection regulations. SPM12, R and extensions are publicly available open-access software. Requests regarding analysis details may be send to Dr. Lammers-Lietz (florian.lammers@charite.de). The probabilistic map of the BFCS used here has been provided by Dr. Laszlo Zaborzsky and may be retrieved from him.

## Declarations

Dr. Maria Heinrich and Dr. Florian Lammers-Lietz are the guarantors who take responsibility for the published work.

## References

[CR1] Adage T, Del Bene F, Fiorentini F, Doornbos RP, Zankl C, Bartley MR, Kungl AJ (2015) PA401, a novel CXCL8-based biologic therapeutic with increased glycosaminoglycan binding, reduces bronchoalveolar lavage neutrophils and systemic inflammatory markers in a murine model of LPS-induced lung inflammation. Cytokine 76(2):433–441. 10.1016/j.cyto.2015.08.006 26303011 10.1016/j.cyto.2015.08.006

[CR2] Anazodo UC, Shoemaker JK, Suskin N, St Lawrence KS (2013) An investigation of changes in regional gray matter volume in cardiovascular disease patients, pre and post cardiovascular rehabilitation. Neuroimage Clin 3:388–395. 10.1016/j.nicl.2013.09.011 24273722 10.1016/j.nicl.2013.09.011 PMC3814972

[CR3] Avram M, Grothe MJ, Meinhold L, Leucht C, Leucht S, Borgwardt S, Brandl F, Sorg C (2021) Lower cholinergic basal forebrain volumes link with cognitive difficulties in schizophrenia. Neuropsychopharmacology 46(13):2320–2329. 10.1038/s41386-021-01070-x 34188186 10.1038/s41386-021-01070-x PMC8580980

[CR4] Ballweg T, White M, Parker M, Casey C, Bo A, Farahbakhsh Z, Kayser A, Blair A, Lindroth H, Pearce RA, Blennow K, Zetterberg H, Lennertz R, Sanders RD (2021) Association between plasma tau and postoperative delirium incidence and severity: a prospective observational study. Br J Anaesth 126(2):458–466. 10.1016/j.bja.2020.08.061 33228978 10.1016/j.bja.2020.08.061 PMC8014913

[CR5] Banks WA, Kastin AJ, Gutierrez EG (1994) Penetration of interleukin-6 across the murine blood-brain barrier. Neurosci Lett 179(1–2):53–56. 10.1016/0304-3940(94)90933-4 7845624 10.1016/0304-3940(94)90933-4

[CR6] Baune BT, Ponath G, Golledge J, Varga G, Arolt V, Rothermundt M, Berger K (2008) Association between IL-8 cytokine and cognitive performance in an elderly general population–the MEMO-Study. Neurobiol Aging 29(6):937–944. 10.1016/j.neurobiolaging.2006.12.003 17207897 10.1016/j.neurobiolaging.2006.12.003

[CR7] Berlot R, Pirtosek Z, Brezovar S, Koritnik B, Teipel SJ, Grothe MJ, Ray NJ (2022) Cholinergic basal forebrain and hippocampal structure influence visuospatial memory in Parkinson’s disease. Brain Imaging Behav 16(1):118–129. 10.1007/s11682-021-00481-0 34176042 10.1007/s11682-021-00481-0

[CR8] Bilusic M, Heery CR, Collins JM, Donahue RN, Palena C, Madan RA, Karzai F, Marte JL, Strauss J, Gatti-Mays ME, Schlom J, Gulley JL (2019) Phase I trial of HuMax-IL8 (BMS-986253), an anti-IL-8 monoclonal antibody, in patients with metastatic or unresectable solid tumors. J Immunother Cancer 7(1):240. 10.1186/s40425-019-0706-x 31488216 10.1186/s40425-019-0706-x PMC6729083

[CR9] Bosancic Z, Spies CD, Müller A, Winterer G, Piper SK, Heinrich M (2022) Association of cholinesterase activities and POD in older adult abdominal surgical patients. BMC Anesthesiol 22(1):293. 10.1186/s12871-022-01826-y 36114455 10.1186/s12871-022-01826-y PMC9479414

[CR10] Butler T, Harvey P, Deshpande A, Tanzi E, Li Y, Tsui W, Silver C, Fischer E, Wang X, Chen J, Rusinek H, Pirraglia E, Osorio RS, Glodzik L, de Leon MJ (2018) Basal forebrain septal nuclei are enlarged in healthy subjects prior to the development of Alzheimer’s disease. Neurobiol Aging 65:201–205. 10.1016/j.neurobiolaging.2018.01.014 29499501 10.1016/j.neurobiolaging.2018.01.014 PMC6413730

[CR11] Casey CP, Lindroth H, Mohanty R, Farahbakhsh Z, Ballweg T, Twadell S, Miller S, Krause B, Prabhakaran V, Blennow K, Zetterberg H, Sanders RD (2020) Postoperative delirium is associated with increased plasma neurofilament light. Brain 143(1):47–54. 10.1093/brain/awz354 31802104 10.1093/brain/awz354 PMC6935744

[CR12] Cavallari M, Dai W, Guttmann CR, Meier DS, Ngo LH, Hshieh TT, Callahan AE, Fong TG, Schmitt E, Dickerson BC, Press DZ, Marcantonio ER, Jones RN, Inouye SK, Alsop DC, Group SS (2016) Neural substrates of vulnerability to postsurgical delirium as revealed by presurgical diffusion MRI. Brain 139(Pt 4):1282–1294. 10.1093/brain/aww010 26920674 10.1093/brain/aww010 PMC5006228

[CR13] Cavedo E, Grothe MJ, Colliot O, Lista S, Chupin M, Dormont D, Houot M, Lehericy S, Teipel S, Dubois B, Hampel H, Hippocampus Study G (2017) Reduced basal forebrain atrophy progression in a randomized Donepezil trial in prodromal Alzheimer’s disease. Sci Rep 7(1):11706. 10.1038/s41598-017-09780-3 28916821 10.1038/s41598-017-09780-3 PMC5601919

[CR14] Conti B, Park LC, Calingasan NY, Kim Y, Kim H, Bae Y, Gibson GE, Joh TH (1999) Cultures of astrocytes and microglia express interleukin 18. Brain Res Mol Brain Res 67(1):46–52. 10.1016/s0169-328x(99)00034-0 10101231 10.1016/s0169-328x(99)00034-0

[CR15] Feinkohl I, Borchers F, Burkhardt S, Krampe H, Kraft A, Speidel S, Kant IMJ, van Montfort SJT, Aarts E, Kruppa J, Slooter A, Winterer G, Pischon T, Spies C (2020) Stability of neuropsychological test performance in older adults serving as normative controls for a study on postoperative cognitive dysfunction. BMC Res Notes 13(1):55. 10.1186/s13104-020-4919-3 32019577 10.1186/s13104-020-4919-3 PMC7001199

[CR16] Field RH, Gossen A, Cunningham C (2012) Prior pathology in the basal forebrain cholinergic system predisposes to inflammation-induced working memory deficits: reconciling inflammatory and cholinergic hypotheses of delirium. J Neurosci 32(18):6288–6294. 10.1523/JNEUROSCI.4673-11.2012 22553034 10.1523/JNEUROSCI.4673-11.2012 PMC3359617

[CR17] Gabay C, Fautrel B, Rech J, Spertini F, Feist E, Kotter I, Hachulla E, Morel J, Schaeverbeke T, Hamidou MA, Martin T, Hellmich B, Lamprecht P, Schulze-Koops H, Courvoisier DS, Sleight A, Schiffrin EJ (2018) Open-label, multicentre, dose-escalating phase II clinical trial on the safety and efficacy of tadekinig alfa (IL-18BP) in adult-onset Still’s disease. Ann Rheum Dis 77(6):840–847. 10.1136/annrheumdis-2017-212608 29472362 10.1136/annrheumdis-2017-212608 PMC5965361

[CR18] Grothe M, Heinsen H, Teipel S (2013) Longitudinal measures of cholinergic forebrain atrophy in the transition from healthy aging to Alzheimer’s disease. Neurobiol Aging 34(4):1210–1220. 10.1016/j.neurobiolaging.2012.10.018 23158764 10.1016/j.neurobiolaging.2012.10.018 PMC4058576

[CR19] Gu Y, Vorburger R, Scarmeas N, Luchsinger JA, Manly JJ, Schupf N, Mayeux R, Brickman AM (2017) Circulating inflammatory biomarkers in relation to brain structural measurements in a non-demented elderly population. Brain Behav Immun 65:150–160. 10.1016/j.bbi.2017.04.022 28457809 10.1016/j.bbi.2017.04.022 PMC5537030

[CR20] Hampel H, Mesulam MM, Cuello AC, Farlow MR, Giacobini E, Grossberg GT, Khachaturian AS, Vergallo A, Cavedo E, Snyder PJ, Khachaturian ZS (2018) The cholinergic system in the pathophysiology and treatment of Alzheimer’s disease. Brain 141(7):1917–1933. 10.1093/brain/awy132 29850777 10.1093/brain/awy132 PMC6022632

[CR21] Heinrich M, Müller A, Lammers-Lietz F, Borchers F, Mörgeli R, Kruppa J, Zacharias N, Winterer G, Slooter AJC, Spies CD (2020) Radiological, Chemical and Pharmacological Cholinergic System Parameters and Neurocognitive Disorders in Older Pre-Surgical Adults. J Gerontol: Series A. 10.1093/gerona/glaa182 10.1093/gerona/glaa182 32710543

[CR22] Heinrich M, Sieg M, Kruppa J, Nurnberg P, Schreier PH, Heilmann-Heimbach S, Hoffmann P, Nothen MM, Janke J, Pischon T, Slooter AJC, Winterer G, Spies CD (2021) Association between genetic variants of the cholinergic system and postoperative delirium and cognitive dysfunction in elderly patients. BMC Med Genomics 14(1):248. 10.1186/s12920-021-01071-1 34674705 10.1186/s12920-021-01071-1 PMC8529799

[CR23] Houdek HM, Larson J, Watt JA, Rosenberger TA (2014) Bacterial lipopolysaccharide induces a dose-dependent activation of neuroglia and loss of basal forebrain cholinergic cells in the rat brain. Inflamm Cell Signal 1(1). 10.14800/ics.47 10.14800/ics.47 PMC4457330 26052539

[CR24] Kant IMJ, de Bresser J, van Montfort SJT, Witkamp TD, Walraad B, Spies CD, Hendrikse J, van Dellen E, Slooter AJC, BioCog C (2023) Postoperative delirium is associated with grey matter brain volume loss. Brain Commun 5(1):fcad013. 10.1093/braincomms/fcad013 10.1093/braincomms/fcad013 PMC9933897 36819940

[CR25] Kilimann I, Hausner L, Fellgiebel A, Filippi M, Wurdemann TJ, Heinsen H, Teipel SJ (2017) Parallel Atrophy of Cortex and Basal Forebrain Cholinergic System in Mild Cognitive Impairment. Cereb Cortex 27(3):1841–1848. 10.1093/cercor/bhw019 26879092 10.1093/cercor/bhw019

[CR26] Kline RP, Pirraglia E, Cheng H, De Santi S, Li Y, Haile M, de Leon MJ, Bekker A, Neuroimaging AD, I. (2012) Surgery and brain atrophy in cognitively normal elderly subjects and subjects diagnosed with mild cognitive impairment. Anesthesiology 116(3):603–612. 10.1097/ALN.0b013e318246ec0b 22293721 10.1097/ALN.0b013e318246ec0b PMC3418798

[CR27] Kline R, Wong E, Haile M, Didehvar S, Farber S, Sacks A, Pirraglia E, de Leon MJ, Bekker A (2016) Peri-Operative Inflammatory Cytokines in Plasma of the Elderly Correlate in Prospective Study with Postoperative Changes in Cognitive Test Scores. Int J Anesthesiol Res 4(8):313–321. 10.19070/2332-2780-1600065 10.19070/2332-2780-1600065 PMC5351884 28317003

[CR28] Knaak C, Vorderwulbecke G, Spies C, Piper SK, Hadzidiakos D, Borchers F, Brockhaus WR, Radtke FM, Lachmann G (2019) C-reactive protein for risk prediction of post-operative delirium and post-operative neurocognitive disorder. Acta Anaesthesiol Scand 63(10):1282–1289. 10.1111/aas.13441 31283835 10.1111/aas.13441

[CR29] Lammers F, Borchers F, Feinkohl I, Hendrikse J, Kant IMJ, Kozma P, Pischon T, Slooter AJC, Spies C, van Montfort SJT, Zacharias N, Zaborszky L, Winterer G, BioCog, c. (2018) Basal forebrain cholinergic system volume is associated with general cognitive ability in the elderly. Neuropsychologia 119:145–156. 10.1016/j.neuropsychologia.2018.08.005 30096414 10.1016/j.neuropsychologia.2018.08.005 PMC6338214

[CR30] Lammers F, Zacharias N, Borchers F, Morgeli R, Spies CD, Winterer G (2020) Functional Connectivity of the Supplementary Motor Network Is Associated with Fried’s Modified Frailty Score in Older Adults. J Gerontol A Biol Sci Med Sci 75(12):2239–2248. 10.1093/gerona/glz297 31900470 10.1093/gerona/glz297

[CR31] Lammers F, Mobascher A, Musso F, Shah NJ, Warbrick T, Zaborszky L, Winterer G (2016) Effects of Ncl. Basalis Meynert volume on the Trail-Making-Test are restricted to the left hemisphere. Brain Behav 6(1):e00421. 10.1002/brb3.421 10.1002/brb3.421 PMC4834944 27110442

[CR32] Lammers-Lietz F, Akyuz L, Feinkohl I, Lachmann C, Pischon T, Volk HD, von Hafen C, Yurek F, Winterer G, Spies CD (2022a) Interleukin 8 in postoperative delirium - Preliminary findings from two studies. Brain Behav Immun Health 20:100419. 10.1016/j.bbih.2022.100419 35141571 10.1016/j.bbih.2022.100419 PMC8814304

[CR33] Lammers-Lietz F, Zacharias N, Morgeli R, Spies CD, Winterer G (2022b) Functional connectivity of the supplementary and presupplementary motor areas in postoperative transition between stages of frailty. J Gerontol A Biol Sci Med Sci. 10.1093/gerona/glac012 35040961 10.1093/gerona/glac012

[CR34] Leonard EJ, Yoshimura T (1990) Neutrophil attractant/activation protein-1 (NAP-1 [interleukin-8]). Am J Respir Cell Mol Biol 2(6):479–486. 10.1165/ajrcmb/2.6.479 2189453 10.1165/ajrcmb/2.6.479

[CR35] Lou AR, Madsen KH, Julian HO, Toft PB, Kjaer TW, Paulson OB, Prause JU, Siebner HR (2013) Postoperative increase in grey matter volume in visual cortex after unilateral cataract surgery. Acta Ophthalmol 91(1):58–65. 10.1111/j.1755-3768.2011.02304.x 22103594 10.1111/j.1755-3768.2011.02304.x

[CR36] Loy R, Taglialatela G, Angelucci L, Heyer D, Perez-Polo R (1994) Regional CNS uptake of blood-borne nerve growth factor. J Neurosci Res 39(3):339–346. 10.1002/jnr.490390311 7869426 10.1002/jnr.490390311

[CR37] Mahler DA, Huang S, Tabrizi M, Bell GM (2004) Efficacy and safety of a monoclonal antibody recognizing interleukin-8 in COPD: a pilot study. Chest 126(3):926–934. 10.1378/chest.126.3.926 15364775 10.1378/chest.126.3.926

[CR38] Malaguarnera L, Motta M, Di Rosa M, Anzaldi M, Malaguarnera M (2006) Interleukin-18 and transforming growth factor-beta 1 plasma levels in Alzheimer’s disease and vascular dementia. Neuropathology 26(4):307–312. 10.1111/j.1440-1789.2006.00701.x 16961066 10.1111/j.1440-1789.2006.00701.x

[CR39] McCarrey AC, Pacheco J, Carlson OD, Egan JM, Thambisetty M, An Y, Ferrucci L, Resnick SM (2014) Interleukin-6 is linked to longitudinal rates of cortical thinning in aging. Transl Neurosci 5(1):1–7. 10.2478/s13380-014-0203-0 27066268 10.2478/s13380-014-0203-0 PMC4824945

[CR40] McKie EA, Reid JL, Mistry PC, DeWall SL, Abberley L, Ambery PD, Gil-Extremera B (2016) A Study to Investigate the Efficacy and Safety of an Anti-Interleukin-18 Monoclonal Antibody in the Treatment of Type 2 Diabetes Mellitus. PLoS ONE 11(3):e0150018. 10.1371/journal.pone.0150018 26930607 10.1371/journal.pone.0150018 PMC4773233

[CR41] Mesulam MM, Mufson EJ, Levey AI, Wainer BH (1983) Cholinergic innervation of cortex by the basal forebrain: cytochemistry and cortical connections of the septal area, diagonal band nuclei, nucleus basalis (substantia innominata), and hypothalamus in the rhesus monkey. J Comp Neurol 214(2):170–197. 10.1002/cne.902140206 6841683 10.1002/cne.902140206

[CR42] Mistry P, Reid J, Pouliquen I, McHugh S, Abberley L, DeWall S, Taylor A, Tong X, Rocha Del Cura M, McKie E (2014) Safety, tolerability, pharmacokinetics, and pharmacodynamics of single-dose antiinterleukin- 18 mAb GSK1070806 in healthy and obese subjects. Int J Clin Pharmacol Ther 52(10):867–879. 10.5414/CP202087 25109413 10.5414/CP202087

[CR43] Muller A, Olbert M, Heymann A, Zahn PK, Plaschke K, von Dossow V, Bitzinger D, Barth E, Meister M, Kranke P, Herrmann C, Wernecke KD, Spies CD (2019) Relevance of peripheral cholinesterase activity on postoperative delirium in adult surgical patients (CESARO): A prospective observational cohort study. Eur J Anaesthesiol 36(2):114–122. 10.1097/EJA.0000000000000888 30431498 10.1097/EJA.0000000000000888

[CR44] Narita M, Tanaka H, Togashi T, Abe S (2005) Cytokines involved in CNS manifestations caused by Mycoplasma pneumoniae. Pediatr Neurol 33(2):105–109. 10.1016/j.pediatrneurol.2005.03.003 16087054 10.1016/j.pediatrneurol.2005.03.003

[CR45] Ojala J, Alafuzoff I, Herukka SK, van Groen T, Tanila H, Pirttila T (2009) Expression of interleukin-18 is increased in the brains of Alzheimer’s disease patients. Neurobiol Aging 30(2):198–209. 10.1016/j.neurobiolaging.2007.06.006 17658666 10.1016/j.neurobiolaging.2007.06.006

[CR46] Pearson RC, Sofroniew MV, Powell TP (1984) Hypertrophy of immunohistochemically identified cholinergic neurons of the basal nucleus of Meynert following ablation of the contralateral cortex in the rat. Brain Res 311(1):194–198. 10.1016/0006-8993(84)91418-5 6488042 10.1016/0006-8993(84)91418-5

[CR47] Pearson RC, Sofroniew MV, Powell TP (1985) Hypertrophy of cholinergic neurones of the rat basal nucleus following section of the corpus callosum. Brain Res 338(2):337–340. 10.1016/0006-8993(85)90164-7 4027599 10.1016/0006-8993(85)90164-7

[CR48] Pearson RC, Sofroniew MV, Powell TP (1987) The cholinergic nuclei of the basal forebrain of the rat: hypertrophy following contralateral cortical damage or section of the corpus callosum. Brain Res 411(2):332–340. 10.1016/0006-8993(87)91085-7 3607437 10.1016/0006-8993(87)91085-7

[CR49] Peter J, Lahr J, Minkova L, Lauer E, Grothe MJ, Teipel S, Kostering L, Kaller CP, Heimbach B, Hull M, Normann C, Nissen C, Reis J, Kloppel S (2016) Contribution of the Cholinergic System to Verbal Memory Performance in Mild Cognitive Impairment. J Alzheimers Dis 53(3):991–1001. 10.3233/JAD-160273 27340852 10.3233/JAD-160273 PMC5008225

[CR50] Prinz M, Hanisch UK (1999) Murine microglial cells produce and respond to interleukin-18. J Neurochem 72(5):2215–2218. 10.1046/j.1471-4159.1999.0722215.x 10217305 10.1046/j.1471-4159.1999.0722215.x

[CR51] Racine AM, Touroutoglou A, Abrantes T, Wong B, Fong TG, Cavallari M, Travison TG, Gou Y, Marcantonio ER, Alsop DC, Jones RN, Inouye SK, Dickerson BC, group, S. s. (2020) Older Patients with Alzheimer’s Disease-Related Cortical Atrophy Who Develop Post-Operative Delirium May Be at Increased Risk of Long-Term Cognitive Decline After Surgery. J Alzheimers Dis 75(1):187–199. 10.3233/JAD-190380 32250290 10.3233/JAD-190380 PMC7304614

[CR52] Rehfeld K, Luders A, Hokelmann A, Lessmann V, Kaufmann J, Brigadski T, Muller P, Muller NG (2018) Dance training is superior to repetitive physical exercise in inducing brain plasticity in the elderly. PLoS ONE 13(7):e0196636. 10.1371/journal.pone.0196636 29995884 10.1371/journal.pone.0196636 PMC6040685

[CR53] Rullmann M, Preusser S, Poppitz S, Heba S, Gousias K, Hoyer J, Schutz T, Dietrich A, Muller K, Hankir MK, Pleger B (2019) Adiposity Related Brain Plasticity Induced by Bariatric Surgery. Front Hum Neurosci 13:290. 10.3389/fnhum.2019.00290 31507395 10.3389/fnhum.2019.00290 PMC6718731

[CR54] Schenning KJ, Murchison CF, Mattek NC, Silbert LC, Kaye JA, Quinn JF (2016) Surgery is associated with ventricular enlargement as well as cognitive and functional decline. Alzheimers Dement 12(5):590–597. 10.1016/j.jalz.2015.10.004 26610898 10.1016/j.jalz.2015.10.004 PMC4861667

[CR55] Spooren A, Kolmus K, Laureys G, Clinckers R, De Keyser J, Haegeman G, Gerlo S (2011) Interleukin-6, a mental cytokine. Brain Res Rev 67(1–2):157–183. 10.1016/j.brainresrev.2011.01.002 21238488 10.1016/j.brainresrev.2011.01.002

[CR56] Sprung J, Kruthiventi SC, Warner DO, Knopman DS, Petersen RC, Mielke MM, Jack CR Jr, Graff-Radford J, Martin DP, Hanson AC, Schroeder DR, Przybelski SA, Schulte PJ, Weingarten TN, Vemuri P (2019) Exposure to surgery under general anaesthesia and brain magnetic resonance imaging changes in older adults. Br J Anaesth 123(6):808–817. 10.1016/j.bja.2019.08.024 31587833 10.1016/j.bja.2019.08.024 PMC6883493

[CR57] Su C, Zhao K, Xia H, Xu Y (2019) Peripheral inflammatory biomarkers in Alzheimer’s disease and mild cognitive impairment: a systematic review and meta-analysis. Psychogeriatrics 19(4):300–309. 10.1111/psyg.12403 30790387 10.1111/psyg.12403

[CR58] Sun YX, Minthon L, Wallmark A, Warkentin S, Blennow K, Janciauskiene S (2003) Inflammatory markers in matched plasma and cerebrospinal fluid from patients with Alzheimer’s disease. Dement Geriatr Cogn Disord 16(3):136–144. 10.1159/000071001 12826739 10.1159/000071001

[CR59] Teipel S, Raiser T, Riedl L, Riederer I, Schroeter ML, Bisenius S, Schneider A, Kornhuber J, Fliessbach K, Spottke A, Grothe MJ, Prudlo J, Kassubek J, Ludolph A, Landwehrmeyer B, Straub S, Otto M, Danek A, group, F. T. s. (2016) Atrophy and structural covariance of the cholinergic basal forebrain in primary progressive aphasia. Cortex 83:124–135. 10.1016/j.cortex.2016.07.004 27509365 10.1016/j.cortex.2016.07.004

[CR60] Wheeler RD, Culhane AC, Hall MD, Pickering-Brown S, Rothwell NJ, Luheshi GN (2000) Detection of the interleukin 18 family in rat brain by RT-PCR. Brain Res Mol Brain Res 77(2):290–293. 10.1016/s0169-328x(00)00069-3 10837926 10.1016/s0169-328x(00)00069-3

[CR61] Whitcroft KL, Fischer J, Han P, Raue C, Bensafi M, Gudziol V, Andrews P, Hummel T (2018) Structural Plasticity of the Primary and Secondary Olfactory cortices: Increased Gray Matter Volume Following Surgical Treatment for Chronic Rhinosinusitis. Neuroscience 395:22–34. 10.1016/j.neuroscience.2018.10.011 30326289 10.1016/j.neuroscience.2018.10.011

[CR62] Winterer G, Androsova G, Bender O, Boraschi D, Borchers F, Dschietzig TB, Feinkohl I, Fletcher P, Gallinat J, Hadzidiakos D, Haynes JD, Heppner F, Hetzer S, Hendrikse J, Ittermann B, Kant IMJ, Kraft A, Krannich A, Krause R, Consortium B (2018) Personalized risk prediction of postoperative cognitive impairment - rationale for the EU-funded BioCog project. Eur Psychiatry 50:34–39. 10.1016/j.eurpsy.2017.10.004 10.1016/j.eurpsy.2017.10.004 29398565

[CR63] Wu C, Wang R, Li X, Chen J (2016) Preoperative Serum MicroRNA-155 Expression Independently Predicts Postoperative Cognitive Dysfunction After Laparoscopic Surgery for Colon Cancer. Med Sci Monit 22:4503–4508. 10.12659/msm.898397 10.12659/MSM.898397 PMC5123778 27872469

[CR64] Yasuda K, Nakanishi K, Tsutsui H (2019) Interleukin-18 in Health and Disease. Int J Mol Sci 20(3). 10.3390/ijms20030649 10.3390/ijms20030649 PMC6387150 30717382

[CR65] Zaborszky L, Hoemke L, Mohlberg H, Schleicher A, Amunts K, Zilles K (2008) Stereotaxic probabilistic maps of the magnocellular cell groups in human basal forebrain. Neuroimage 42(3):1127–1141. 10.1016/j.neuroimage.2008.05.055 18585468 10.1016/j.neuroimage.2008.05.055 PMC2577158

