# Supplementary material for: Perioperative Levels of IL8 and IL18, but not IL6, are Associated with Nucleus Basalis Magnocellularis Atrophy Three Months after Surgery
Source: J Neuroimmune Pharmacol. 2024 Mar 14;19(1):10. doi: 10.1007/s11481-024-10110-4 (PMC10940494; doi:10.1007/s11481-024-10110-4)
Supplement: Supplementary file 1 — Supplementary file1 (PDF 6943 KB) [file 11481_2024_10110_MOESM1_ESM.pdf]

Supplement to:

# Perioperative levels of IL8 and IL18, but not IL6, are associated with Nucleus basalis magnocellularis atrophy three months after surgery

Maria Heinrich[1,2] (ORCID: 0000-0002-4530-7343), Claudia Spies (ORCID: 0000-0002-1062-0495)[1], Friedrich Borchers[1] (ORCID: 0000-0001-7290-2271), Insa Feinkohl[3,4] (ORCID: 0000-0002-0307-1862), Tobias Pischon[4-7] (ORCID: 0000-0003-1568-767X), Arjen Slooter[8-12] (ORCID: 0000-0003-0804-8378), Clarissa von Haefen[1] (ORCID: 0000-0001-5740-1794), Norman Zacharias[1,3,14] (ORCID: 0000-0001-5794-5716), Georg Winterer[1,13,14] (ORCID: 0000-0002-3610-5029), Florian Lammers-Lietz[1] (ORCID: 0000-0002-7371-3947) on behalf of the BioCog consortium

1 Charité–Universitätsmedizin Berlin, corporate member of Freie Universität Berlin and Humboldt-Universität zu Berlin, Department of Anesthesiology and Intensive Care Medicine Augustenburger Platz 1, 13353 Berlin, Germany

2 Berlin Institute of Health at Charité – Universitätsmedizin Berlin, Berlin, Germany

3 Faculty of Health/Department of Medicine at Witten/Herdecke University, Witten/Herdecke, Germany

4 Molecular Epidemiology Research Group, Max-Delbrück-Center for Molecular Medicine in the Helmholtz Association (MDC), Berlin, Germany

5 Biobank Technology Platform, Max-Delbrück-Center for Molecular Medicine in the Helmholtz Association (MDC), Berlin, Germany

6 Charité–Universitätsmedizin Berlin, corporate member of Freie Universität Berlin and Humboldt-Universität zu Berlin

7 Berlin Institute of Health at Charité – Universitätsmedizin Berlin, Core Facility Biobank, Berlin, Germany

8 Department of Intensive Care Medicine and Brain Center, University Medical Center Utrecht, Utrecht University, Utrecht, the Netherlands

9 Department of Psychiatry, University Medical Center Utrecht, Utrecht University, Utrecht, the Netherlands

11 UMC Utrecht Brain Center, Utrecht University, Utrecht, the Netherlands

12 Department of Neurology, UZ Brussel and Vrije Universiteit Brussel, Brussels, Belgium

13 Pharmaimage Biomarker Solutions GmbH, Berlin, Germany

14 PI Health Solutions GmbH, Berlin Germany

*CORRESPONDING AUTHOR: Florian Lammers-Lietz, Dept. of Anesthesiology and Intensive Care Medicine, Charité-Universitätsmedizin Berlin, Augustenburger Platz 1, 13353 Berlin (internal address: Mittelallee 3), e-mail: florian.lammers@charite.de*

## Supplementary A: Exclusion criteria

Exclusion criteria were refusal of the patient, MMSE score ≤23 points, neuropsychiatric morbidity, centrally acting medication, or sensory impairment interfering with neurocognitive testing or MRI, homelessness, or unavailability of the patient for follow-up assessments, simultaneous participation in another prospective interventional clinical trial, and accommodation in an institution due to an official or judicial order.

# Supplementary B: Quantile regression

We repeated analyses using quantile regression for two reasons: First, we noted that one patient showed an unexpected NBM volume gain after surgery. Since quantile regression is robust against outliers in the dependent variable, we compared regression coefficients from OLS regression and regression to the 0.5$^{th}$ quantile to assure that the outlier did not affect our conclusions. Second, quantile regression gives a more comprehensive overview on changes of the distribution of the dependent variable: Here, we were concerned that additionally to NBM atrophy rate, interindividual variability between atrophy rates would be higher in patients with a high level of inflammation.

## Methods

Quantile regression was conducted using the quantreg package for R. Standard errors were calculated from 1 000 000 bootstrap samples. Treatment of independent variables in quantile regression and ordinary least squares (OLS) models was identical, including the same kind of variable transformation to allow direct comparison of regression coefficients.

Since OLS regression determines the central tendency of an association between interleukin levels and NBM atrophy rate, model coefficients from OLS regression were compared to coefficients from quantile regression at the 0.5$^{th}$ quantile as an overall indicator for the central tendency. Additionally, we visualized quantile regression coefficients for a wide range of quantiles (0.1$^{st}$ to 0.9$^{th}$ quantile in intervals of 0.05) to assess changes in the distribution of NBM atrophy rates depending on the interleukin levels.

## Results

Supplementary table S1 gives all regression coefficients for perioperative interleukin levels and changes. Supplementary figure S1 visualizes differences between OLS and quantile regression for models for models of postoperative interleukin levels, and supplementary figure S2 does so for models of acute on chronic effects. Generally, regression coefficients at the 0.5$^{th}$ quantile were comparable to results from OLS regression. With exception of the association between preoperative IL8 levels and NBM atrophy which achieved significance in OLS, both approaches led to the same conclusions (of note, the analysis of IL8 levels was not contaminated by outliers, and hence, OLS regression results should be reliable).

Inspection of regression coefficients over a wide range of quantiles showed that regression coefficient at more extreme quantiles (i.e., 0.1$^{st}$ or 0.9$^{th}$) differed in some cases from regression coefficients at central quantiles (i.e., 0.5$^{th}$), but confidence intervals of coefficients at different quantiles were generally wide and overlapping. At a qualitative level, no general pattern could be observed.

## Comment

The rationale for supplementary quantile regression analyses was one extreme and implausible value in the dependent variable which may have biased OLS regression. Quantile regression is less sensitive to such outliers, and hence, the replication of regression coefficients confirms that our results from OLS regression were not distorted. Analysis of more extreme quantiles did not support our hypothesis that inflammation influences the distribution of NBM atrophy beyond its central tendency.

**Supplementary table S1.** Quantile regression results for association of postoperative interleukin levels (model 1-3) and models of acute on chronic effects (model 4-6).

| Model | Dependent variable | Regression coefficient at 0.5$^{th}$ quantile (95% confidence interval) | p |
|---|---|---|---|
| **1** | Postoperative IL6 | 0.0877 (-0.0101; 0.1856) | 0.080 |
| **2** | Postoperative IL8 | 1.027 (0.112; 1.943) | 0.0305 |
| **3** | Postoperative IL18 | 1.150 (0.454; 1.847) | 0.0014 |
| **4** | Preoperative IL6 | 0.0416 (-0.1628; 0.2460) | 0.7 |
| | Postoperative IL6 increase | 0.0998 (-0.0032; 0.2028) | 0.058 |
| **5** | Preoperative IL8 | 2.251 (-0.272; 4.77) | 0.092 |
| | Postoperative IL8 increase | 1.047 (-0.455; 2.55) | 0.18 |
| **6** | Preoperative IL18 | 1.118 (0.203; 2.033) | 0.0175 |
| | Postoperative IL18 increase | 0.798 (-0.319; 1.916) | 0.16 |

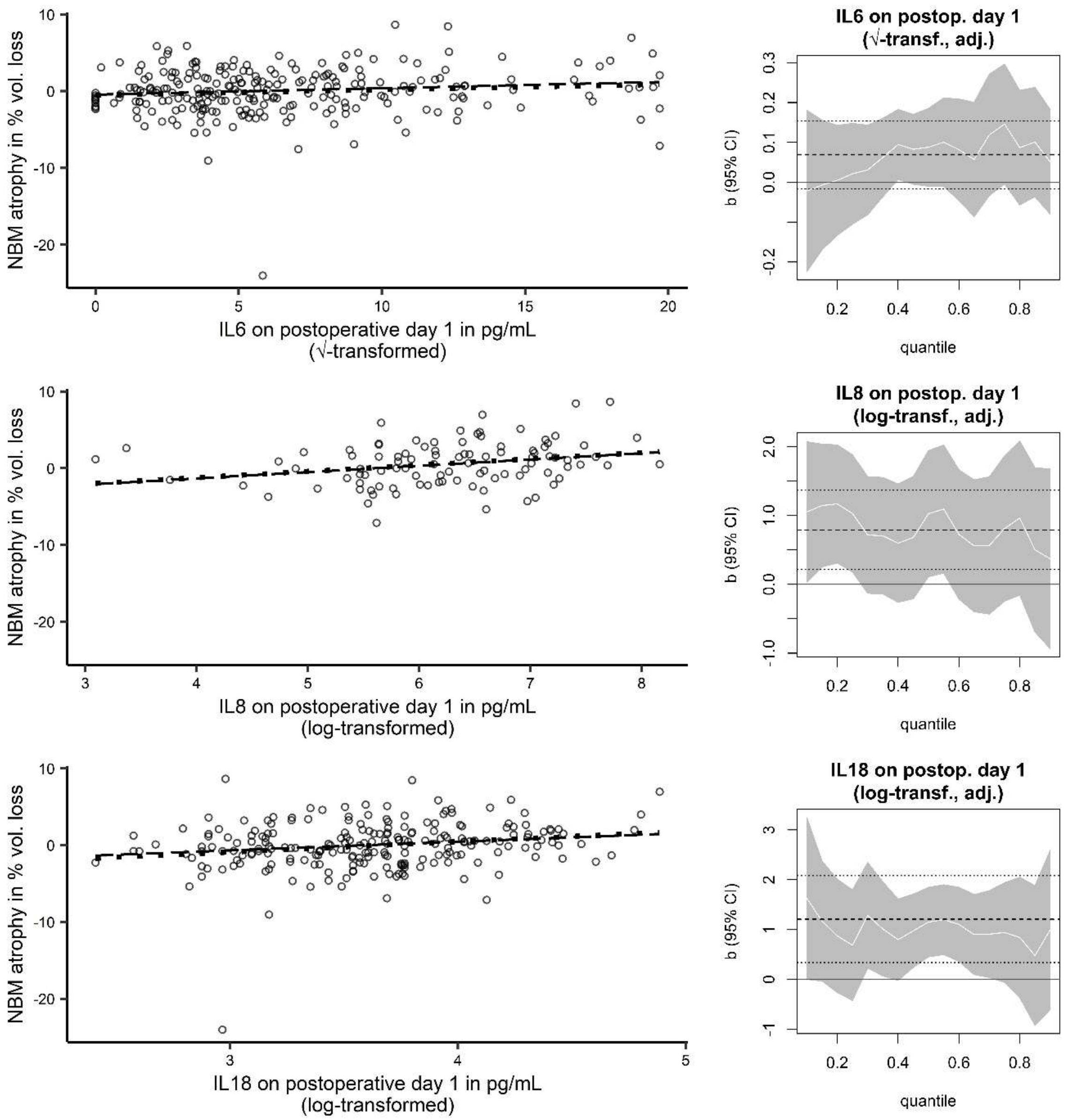


**Supplementary figure S1.** Comparison of OLS and quantile regression models. The left column compares OLS regression slopes (dotted line) and quantile regression slopes (dashed lines) for the association of NBM atrophy (ordinate) and levels of IL6 (top), IL8 (middle) and IL18 (bottom) on postoperative day 1 (abscissa). Data points have not been residualized. Of note, a positive value indicates volume loss, whereas a negative value reflects a gain of volume in comparison to baseline. Correspondingly, OLS regression and quantile regression coefficient from multiple regression models adjusted for age, sex, MMSE and MRI scanner are compared in the right column: Coefficients (b) are given on the ordinate. The coefficient from OLS regression is given as a horizontal dashed black line with 95% confidence interval (CI) indicated by two dotted lines. Coefficients from quantile regression were calculated for 17 percentiles (0.1st to 0.9th in 0.05 intervals) and are displayed as a white line graph with corresponding 95% CI (shaded grey area). The respective quantile is given on the abscissa. For orientation, a solid black line at b=0 (no association) is given. Abbreviations: adj. – adjusted, NBM – nucleus basalis magnocellularis (of Meynert), transf. – transformed, vol. – volume

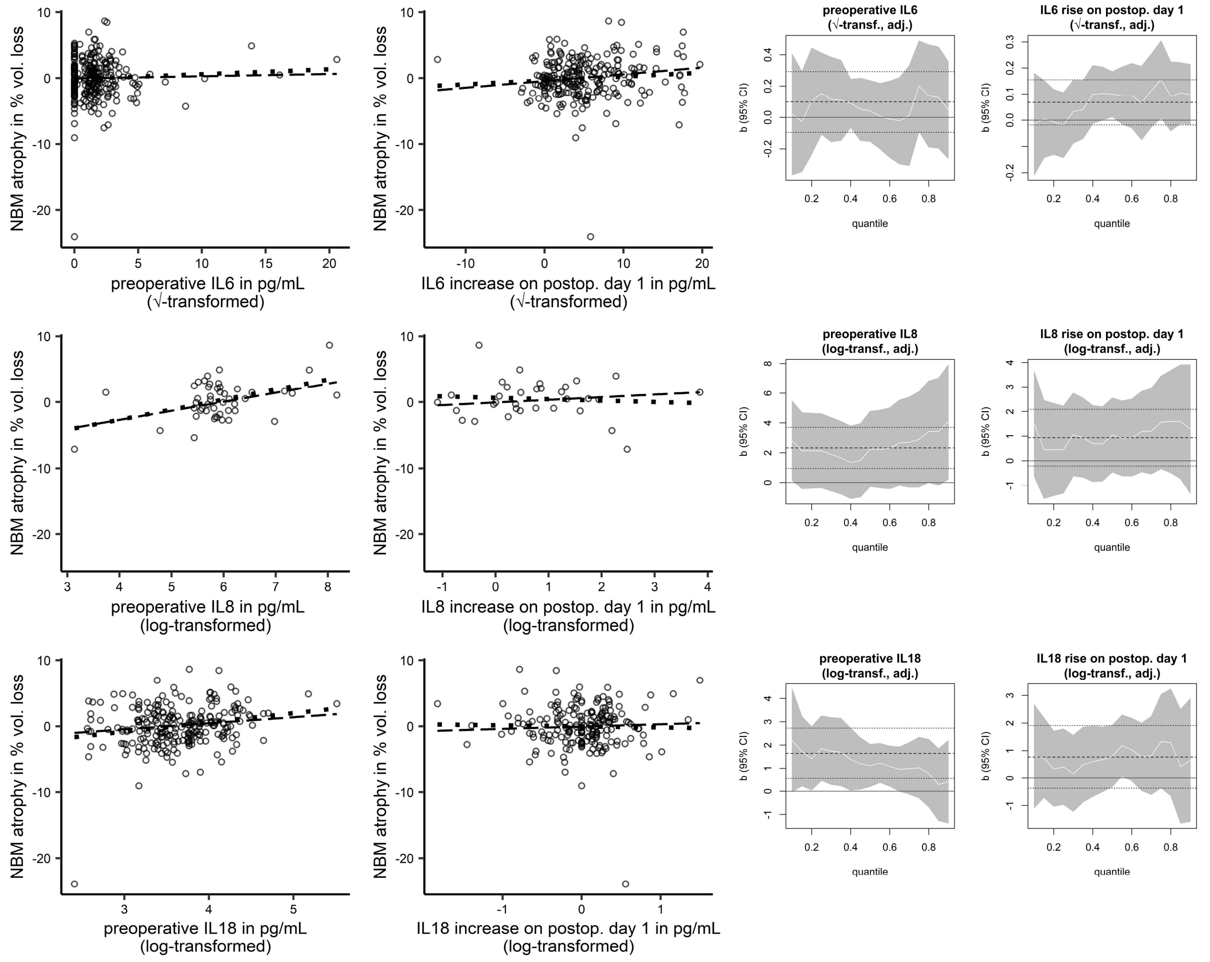


**Supplementary figure S2.** Comparison of coefficients from OLS and quantile regression. Scatter plots show OLS regression slopes (dotted line) and quantile regression slopes (dashed lines) for the association of NBM atrophy (ordinate) and levels of IL6 (top), IL8 (middle) and IL18 (bottom) for unrezidualized data. In the left column, preoperative interleukin levels are plotted against atrophy and in the right column, the increase of interleukin levels from baseline to postoperative day 1 is given on the abscissa. Positive values indicate an increase, in patients with negative values, interleukin levels dropped on the first postoperative day. Plots in the two columns on the right compare OLS regression coefficients with coefficients from quantile regression from multiple regression models including age, sex, MMSE and MRI scanner as nuisance variables for preoperative interleukin levels (first column from left) and interleukin increase on the first postoperative day (farthest right column). OLS regression coefficient is given as a dashed black line (dotted lines: 95% CI). Quantile regression coefficients are given as a white line graph (shaded area: 95% CI). Abbreviations: adj. – adjusted, NBM – nucleus basalis magnocellularis (of Meynert), transf. – transformed, vol. – volume

# Supplementary C: Comparative analysis

We repeated the analysis using global brain atrophy instead of NBM atrophy to assess if the association was specific for cholinergic cell regions. We further compared models including interleukin levels with a model which included duration of anesthesia and ICU admission as independent variables instead, as we considered it a more global, but unspecific surrogate parameter for surgical trauma. Finally, we report associations of preoperative interleukin levels with preoperative NBM volume.

## Methods

MR images were partitioned into grey and white matter as well as cerebrospinal fluid using the SPM12 segmentation routine (http://www.fil.ion.ucl.ac.uk/spm/software/spm12/) in a MATLAB environment (The Mathworks. Inc. Natick. MA). A voxel was assigned to a tissue partition if a ≥50% probability for this voxel to belong to this partition was given. Total brain volume was calculated as the sum of voxel in grey and white matter partitions.

For the analysis of anesthesia duration, type of surgery and ICU admission, all patients with longitudinal MRI were considered. Type of surgery distinguished between peripheral surgery and procedures opening one of the body cavities. The final sample size for these analyses is hence N>240 and reported with results. A sample description is given in table S2.

Analysis of associations between interleukin levels with global brain atrophy were conducted analogously to analyses of NBM atrophy: Brain atrophy (tissue loss in %) was treated as the dependent in variable in three models, each including concentration of IL6, IL8 or IL18 as the independent variable of interest.

Analysis of associations between NBM atrophy with three clinical surrogate parameters for surgical trauma were conducted analogously to analyses of postoperative interleukin levels. NBM atrophy was treated as the dependent variable in three different models, each containing either ICU admission, type of surgery or duration of anesthesia as the independent variable of interest instead of interleukin concentrations. Anesthesia duration underwent logarithmic transformation prior to analysis to approximate a normal distribution.

Analyses of preoperative interleukin levels and preoperative NBM volume were conducted analogously to analyses of NBM atrophy. NBM volume was treated as the dependent variable in three different models, each containing either preoperative IL6, IL8 or IL18 levels as the independent variable of interest instead of interleukin concentrations. We repeated the analysis with additional adjustment for intracranial volume as a measure of past maximum brain volume to get a more precise measure of NBM atrophy at baseline.

All models were adjusted for age, sex baseline MMSE score and up to three dummy variables for MR scanner.

## Results

Brain atrophy was not significantly associated with IL6 (b=0.0474 [-0.233; 0.18181], p=0.19, partial $R^2$=0.007), IL8 (b=0.181 [-0.088; 0.452], p=0.18, partial $R^2$=0.020) nor IL18 (b=0.459 [-0.246; 1.164], p=0.20, partial $R^2$=0.008). Notably, all regression slopes and effect sizes were lower in models of global brain atrophy compared to NBM atrophy.

Supplementary figures S3-S5 describe associations of interleukin levels with clinical surrogate parameters for surgical trauma. Postoperative IL6 levels, but none of the other interleukins was associated significantly with all three surrogate parameters. Neither duration of anesthesia (N=300, b=0.428 [-0.130; 0.987], p=0.13, partial $R^2$=0.008), nor surgical procedures opening body cavities

(N=306, b=0.022 [-0.624; 0.669], p=0.9, partial $R^2<0.001$) were associated with NBM atrophy. NBM atrophy was not significantly higher in patients with ICU admission (N=308, b=0.837 [-0.054; 1.727], p=0.065, partial $R^2=0.011$).

Preoperative concentrations of IL6, (N=265, b=1.01 [-7.20; 9.21], p=0.8, partial $R^2<0.001$), IL8 (N=47, b=10.4 [-43.3; 64.0], p=0.7, partial $R^2=0.004$) and IL18 (b=26.0 [-14.1; 66.1], p=0.20, partial $R^2=0.007$) were not significantly associated with preoperative NBM volume. Results were not altered by adding intracranial volume as an independent variable into the models to adjust for past maximum brain volume.

## Comment

Overall brain volume was not significantly affected by postoperative interleukin levels, suggesting that cholinergic cells are particularly vulnerable to systemic inflammation.

We considered duration of surgery as well as the need for postoperative intensive care as additional measures for surgical trauma, which should be related to the extent of the postoperative immune response. We observed no association of these two measures with NBM atrophy, which supports our conclusion that preoperative inflammation may be of higher relevance than the inflammatory response to surgical trauma.

Our negative finding for association between preoperative interleukin levels and NBM volume do not support the hypothesis of a general association between chronic inflammation and NBM atrophy independent of surgery. However, these results should be interpreted with caution as preoperatively measured interleukin levels probably do not reflect past levels. To the contrary, preoperative interleukin levels are likely to be influenced by pathologies for which patients need to undergo surgery.

**Supplementary table S2.** A description of all 309 patients with longitudinal MRI which were included in the comparative analysis of duration of anesthesia and ICU admission. Continuous variables are described as median, 25th-75th percentile and categorical variables as absolute frequency (relative frequency in %).

| | | |
|---|---|---|
| Age (y) | | 71 (68-76) |
| Women | | 105 (34%) |
| Body mass index (kg/m²) | | 26.3 (24.1-28.6) |
| MMSE (p) | | 29 (28-30) |
| ASA physical score | I | 23 (7%) |
| | II | 208 (67%) |
| | III | 78 (25%) |
| Malignant disease | | 68 (22%) |
| CCI | | 1 (0-2) |
| Preoperative CRP (mg/L) | | 2.3 (1.0-5.9) |
| Postoperative CRP (mg/L) | | 28.8 (4.8-54.3) |
| Preoperative leukocytes ($nL^{-1}$) | | 6.0 (4.9-7.2) |
| Postoperative leukocytes ($nL^{-1}$) | | 9.3 (7.5-11.4) |
| Regional anesthesia | | 24 (8%) |
| Combined regional/general anesthesia | | 42 (14%) |
| Duration of anesthesia (min) | | 195 (117-267) |
| Intracavitary surgery | | 136 (44%) |
| ICU admission | | 57 (18%) |
| Length of hospital stay (d) | | 4 (2-8) |
| Preoperative NBM volume (cm³) | | 1.76 (1.64-1.88) |
| NBM atrophy (%) | | 0.0 (-1.8-1.7) |
| Preoperative brain volume (cm³) | | 1010 (935-1094) |
| Brain atrophy (%) | | 0.4% (-0.3%-1.3%) |

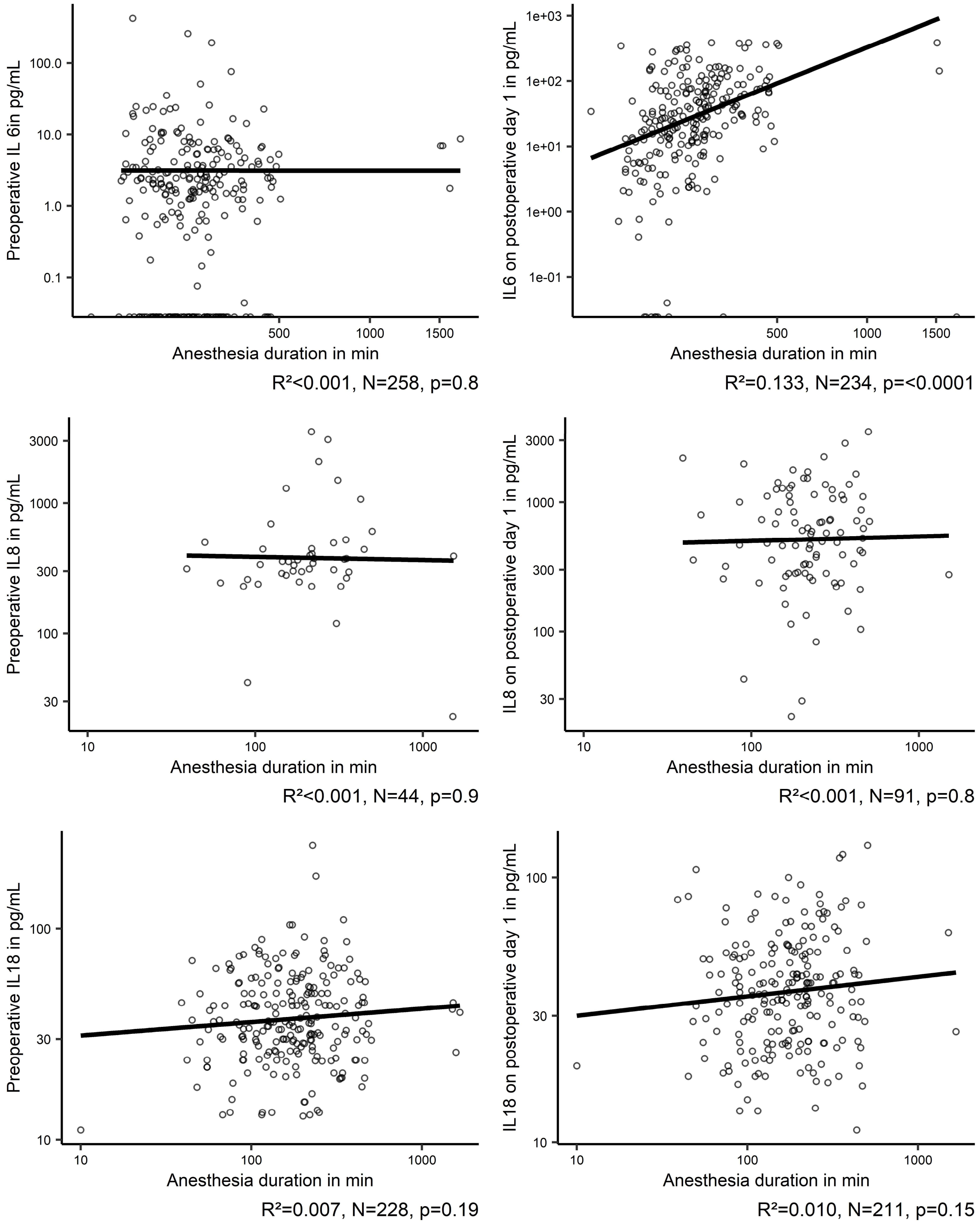


**Supplementary figure S3.** Associations of duration of anesthesia with pre- and postoperative interleukin levels. IL8, IL18 and duration are shown on log-transformed scales, whereas IL6 is given on a square root-transformed scale. $R^2$ and p-value have been derived from simple Pearson’s correlation analyses of transformed variables

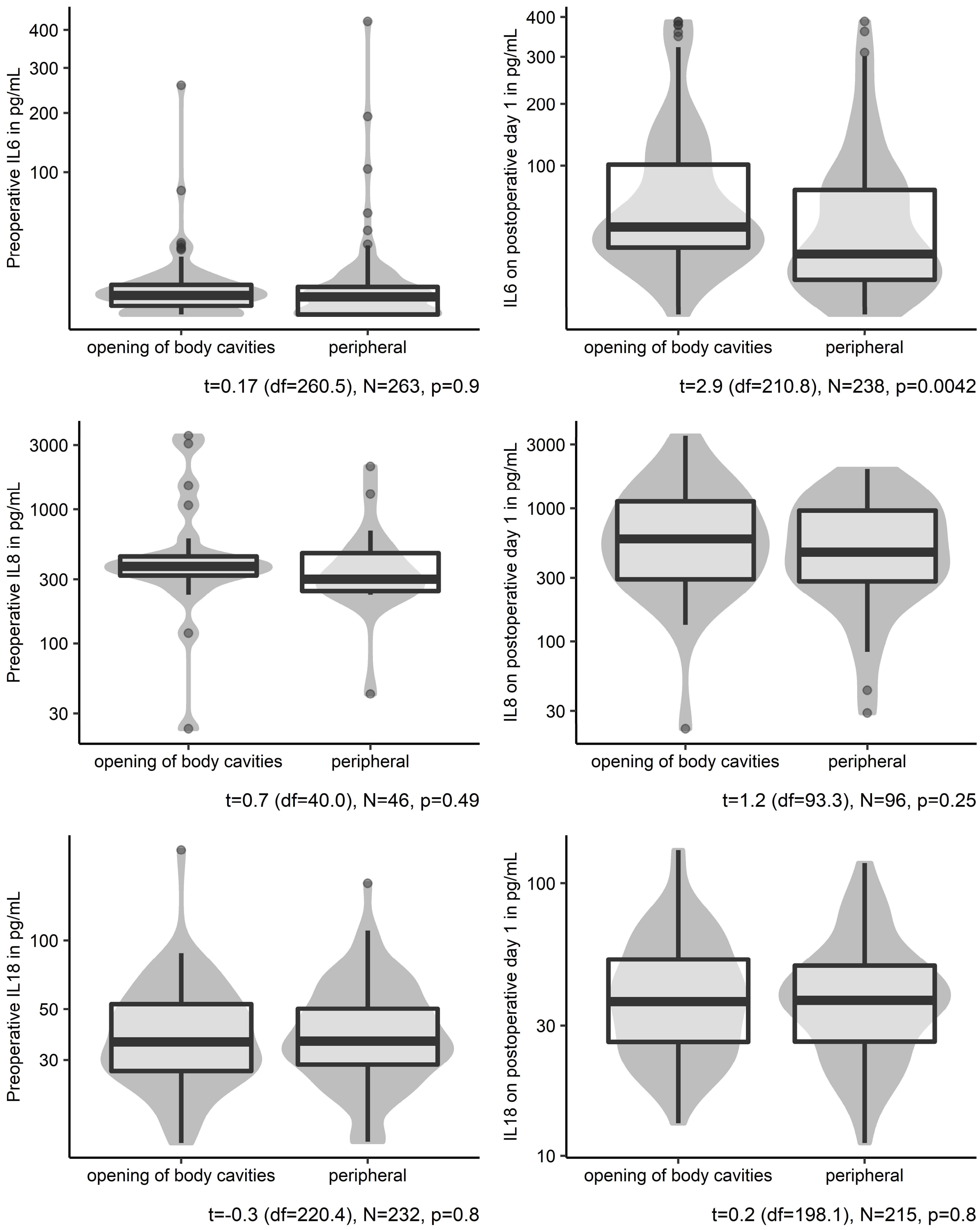


**Supplementary figure S4.** Associations of surgical procedures with pre- and postoperative interleukin levels as violin-plots with overlapping box plots. IL8 and IL18 are shown on log-transformed scales, whereas IL6 is given on a square root-transformed scale. t- and p-values have been derived from simple Welch's two-samples t-test adjusted for unequal variances. The abbreviation "df" refers to the number of degrees of freedom

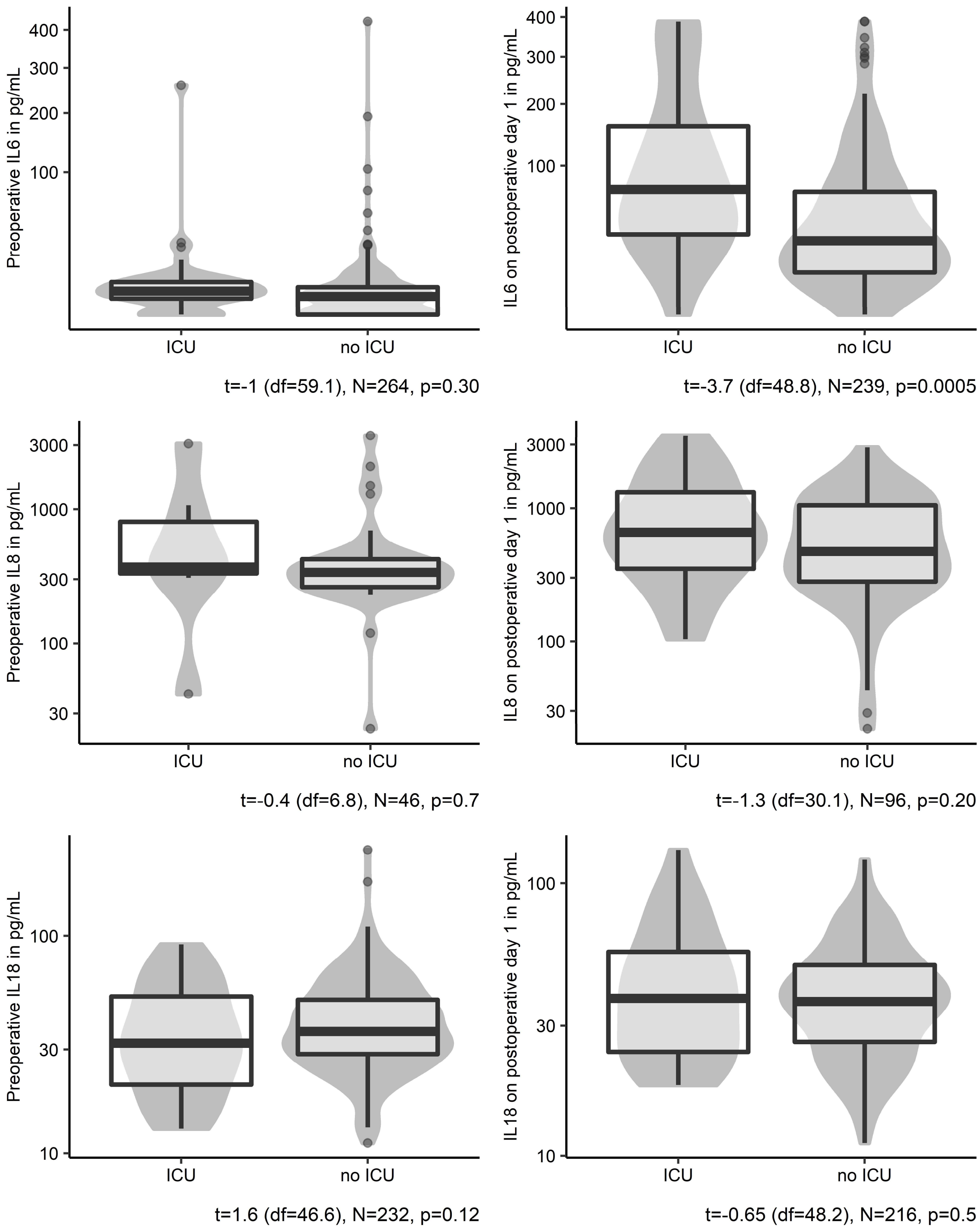


**Supplementary figure S5.** Pre- and postoperative interleukin levels as violin-plots with overlapping box plots in patients with- and without ICU admission. IL8 and IL18 are shown on log-transformed scales, whereas IL6 is given on a square root-transformed scale. t- and p-values have been derived from simple Welch's two-samples t-test adjusted for unequal variances. The abbreviation "df" refers to the number of degrees of freedom, and "ICU" refers to intensive care unit

# Supplementary D: Multicollinearity analysis in models of acute on chronic effects

As shown in figure 2D, there was a negative correlation of preoperative interleukin levels and postoperative change. Hence, both parameters were not independent, leading possibly to collinearity and hampering model parameter estimation.

## Methods

We calculated the variance inflation factor (VIF) for both perioperative change in IL concentrations and preoperative IL levels in each model of NBM atrophy to assess problematic collinearity. We further re-analyzed the data including only preoperative levels or perioperative change as parameters to see if this might affect our results. VIFs below 5 were considered not critical.

## Results

VIF were generally acceptable for IL6 (VIF=1.1 for both parameters), IL8 (2.0 for both parameters) and IL18 (1.4 for both parameters). Supplementary analyses of models including either preoperative values or the postoperative increase did not alter our conclusions (supplementary table S3).

**Supplementary table S3.** Supplementary models of NBM atrophy for analysis of acute on chronic effects. Each model includes only one interleukin-related variable, either preoperative values (models 7-8, 10-11 and 13-14) or the perioperative change (models 9, 12 and 15). The note “all” refers to the inclusion of all patient datasets with preoperative interleukin measurements, regardless of postoperative assessments, as we considered missing postoperative data a possible source of bias.

| Model | Independent variable | N | OLS Regression coefficient (95% confidence interval) | p | Partial $R^2$ |
|---|---|---|---|---|---|
| **7** | Preoperative IL6 | 239 | 0.0535 (-0.1331; 0.2401) | 0.57 | 0.001 |
| **8** | Preoperative IL6 (all) | 265 | 0.0966 (-0.0725; 0.2657) | 0.26 | 0.005 |
| **9** | Perioperative IL6 change | 239 | 0.0553 (-0.0278; 0.1384) | 0.19 | 0.007 |
| **10** | Preoperative IL8 | 33 | 2.040 (0.649; 3.432) | 0.0062* | 0.240 |
| **11** | Preoperative IL8 (all) | 47 | 1.698 (0.769; 2.626) | 0.0006* | 0.250 |
| **12** | Perioperative IL8 change | 33 | -0.338 (-1.402; 0.725) | 0.52 | 0.016 |
| **13** | Preoperative IL18 | 199 | 1.180 (-0.143; 2.502) | 0.080 | 0.026 |
| **14** | Preoperative IL18 (all) | 234 | 1.073 (0.233; 1.913) | 0.0125* | 0.027 |
| **15** | Perioperative IL18 change | 199 | -0.093 (-1.108; 0.921) | 0.9 | <0.001 |

## Supplementary E: Reference values for volumetric parameters

Longitudinal MRI reference data were available for 19 (40%) female and 29 (60%) male participants with a median age of 71 years (IQR: 67-74y, min-max: 65-82y) and a median MMSE of 29 points (IQR: 28-30, min-max: 24-30).

**Supplementary table S4.** Volumetric data in the reference group.

| | Median | IQR |
|---|---|---|
| **Baseline NBM volume ($cm^3$)** | 1.8 | 1.6-1.9 |
| **NBM atrophy (%)** | -0.1 | -1.2-0.8 |
| **Baseline brain volume ($cm^3$)** | 1056 | 949-1126 |
| **Brain atrophy (%)** | 0.0 | -0.9-0.5 |

## Supplementary F: Associations with cognitive outcomes

**Supplementary table S5.** Associations of NBM atrophy (volume loss in %) with cognitive performance change.

| | N (df) | B (95% CI) | p | Partial $R^2$ |
|---|---|---|---|---|
| **SRT latency (ms)[a]** | 284 (8, 275) | -0.961 (-4.337; 2.414) | 0.6 | 0.001 |
| **VRM free recall (items)[b]** | 284 (8, 275) | 0.008 (-0.048; 0.064) | 0.8 | <0.001 |
| **SSP span length (items)[c]** | 284 (8, 275) | 0.000 (-0.031; 0.031) | 1 | <0.001 |
| **PAL memory score (items)[d]** | 280 (8, 271) | -0.125 (-0.263; 0.014) | 0.078 | 0.011 |
| **GPT completion time (s)[e]** | 210 (8, 201) | 0.400 (-0.326; 1.126) | 0.28 | 0.006 |
| **TMT-B completion time (s)[f]** | 284 (8, 275) | -0.855 (-2.135; 0.425) | 0.19 | 0.009 |

Regression analyses of postoperative cognitive function on preoperative cognitive function to derive perioperative cognitive change were done in the sample of N=301 patients with MRI and cognitive data. Index letters refer to a summary of the regression models: [a] intercept= 181.44, B=0.45, $R^2$=0.19; [b] intercept=3.401, B=0.450, $R^2$=0.224; [c] intercept=0.399, B=0.050, $R^2$=0.18; [d] intercept=7.803, B=0.503, $R^2$=0.26; [e] intercept=29.491, B=0.659, $R^2$=0.50; [f] intercept=42.128, B=0.521, $R^2$=0.39

**Supplementary table S6.** Associations of postoperative NBM volume (in $cm^3$) with postoperative cognitive performance.

| | N[a] (df) | B (95% CI) | p | Partial $R^2$ |
|---|---|---|---|---|
| **SRT latency (ms)** | 286 (8, 277) | -0.038 (-0.230; 0.153) | 0.7 | <0.001 |
| **VRM free recall (items)** | 287 (8, 278) | 1.879 (0.700; 3.057) | 0.0019* | 0.034 |
| **SSP span length (items)** | 286 (8, 277) | 0.464 (-0.184; 1.112) | 0.16 | 0.007 |
| **PAL memory score (items)** | 283 (8,274) | 2.775 (-0.220; 5.770) | 0.069 | 0.012 |
| **GPT completion time (s)** | 283 (8, 274) | -0.076 (-0.228; 0.076) | 0.33 | 0.003 |
| **TMT-B completion time (s)** | 273 (8, 264) | -0.264 (-0.519; -0.010) | 0.0417* | 0.016 |

[a] Higher sample sizes in the analysis of postoperative cross-sectional associations between NBM volume and postoperative cognitive performance are related to a minority of patients who only underwent postoperative MRI or did not complete cognitive testing.

## Supplementary G: Assessment of selection bias

From inspection of demographic and clinical sample characteristics it was suspected that the smaller sample of patients with IL8 data had higher morbidity and underwent more extensive procedures compared to the samples with IL6 and IL18 data. This was suspected to introduce selection bias to the analysis of this subsample. We expected stronger associations of IL18 and IL6 with NBM atrophy in the smaller sample if selection bias was to be accounted for the strong association of postoperative IL8 and NBM atrophy.

METHODS: We repeated our analyses in a sample of 95 patients who provided data for all three interleukins on the first postoperative day.

To assess the influence of the multicenter study design on selection bias we visualized the distribution of missing data with respect to study center using the naniar package for R.

RESULTS: Levels of IL6 and IL18 on the first postoperative day tended to be higher in the subsample with complete interleukin assessments. Regression coefficients for IL6 and IL18 were lower when analyzing the subsample with complete interleukin assessments, yielding an insignificant association of IL18 and NBM atrophy. Figures S6 and S7 give an overview of the distribution of missing data.

COMMENT: We observed that the association of IL18 and NBM atrophy was altered by selecting patients with complete interleukin assessments. This might be due to a ceiling effect in patients with more severe inflammation after more extensive surgery. Although these results show that the associations of interleukin levels and NBM atrophy are sensitive to sample selection, they do not suggest that the effect size estimation for IL8 and NBM atrophy was over-estimated, since effect sizes for IL6 and IL18 tended to be smaller in this selected subsample.

An association of missing data interleukin measurements with one study center was observed but did not account for missing IL8 data in particular.

**Supplementary table S7.** Interleukin levels on the first postoperative day in the subsample of 95 patients with three interleukin measurements.

| Model | Median | Interquartile range | Minimum-maximum range |
|---|---|---|---|
| **IL6** | 76 | 34-154 | 2-388 |
| **IL8** | 578 | 282-1087 | 22-3508 |
| **IL18 (pg/mL)** | 43 | 34-65 | 17-132 |

**Supplementary table S8.** Supplementary models of associations of NBM atrophy with postoperative interleukin levels in 95 patients who provided measurements for all three parameters.

| Model | Independent variable | N | OLS Regression coefficient (95% confidence interval) | p | Partial $R^2$ |
|---|---|---|---|---|---|
| **16** | Postoperative IL6 | 95 | 0.015 (-0.100; 0.130) | 0.8 | <0.001 |
| **17** | Postoperative IL8 | 95 | 0.810 (0.226; 1.394) | 0.007* | 0.079 |
| **18** | Postoperative IL18 | 95 | 0.712 (-0.533; 1.957) | 0.26 | 0.014 |

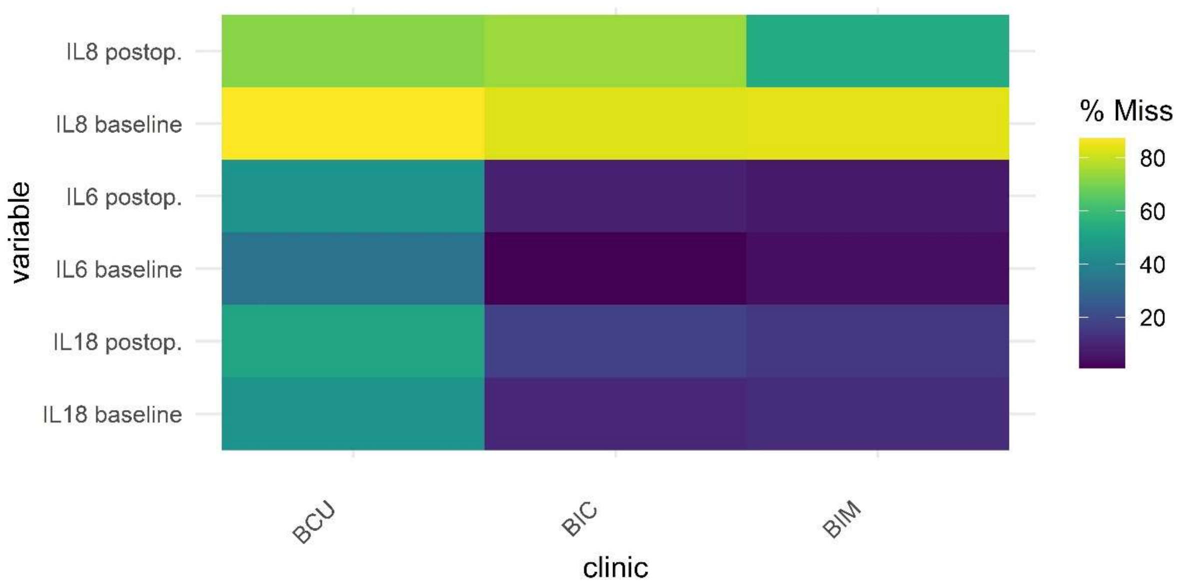


**Supplementary figure S6.** Amount of missing interleukin assessments among N=309 patients with longitudinal MRI data. BCU refers to the study site in Utrecht, Netherlands, whereas BIC and BIM indicate two university hospitals in Berlin.

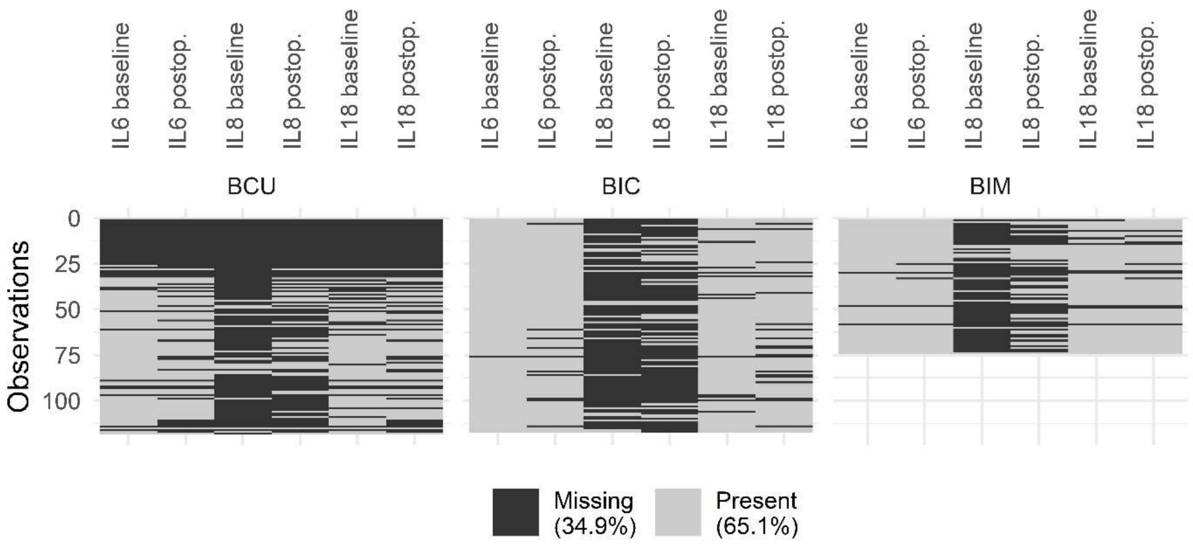


**Supplementary figure S7.** Distribution of missing data during the BioCog study. Observations have been arranged in order of the enrollment of patients, revealing a cluster of missing values at the beginning of study at one study site (BCU, Utrecht).
